# B cell-activating factor (BAFF) from dendritic cells, monocytes and neutrophils is required for B cell maturation and autoantibody production in SLE-like autoimmune disease

**DOI:** 10.3389/fimmu.2023.1050528

**Published:** 2023-02-27

**Authors:** Daniela Giordano, Runa Kuley, Kevin E. Draves, Keith B. Elkon, Natalia V. Giltiay, Edward A. Clark

**Affiliations:** ^1^ Department of Medicine, Division of Rheumatology, University of Washington, Seattle, WA, United States; ^2^ Department of Microbiology, University of Washington, Seattle, WA, United States; ^3^ Department of Immunology, University of Washington, Seattle, WA, United States

**Keywords:** BAFF, autoimmunity, SLE, B cells, autoantibodies, dendritic cells, monocytes, neutrophils

## Abstract

**Purpose and methods:**

B cell-activating factor (BAFF) contributes to the pathogenesis of autoimmune diseases including systemic lupus erythematosus (SLE). Although several anti-BAFF Abs and derivatives have been developed for the treatment of SLE, the specific sources of BAFF that sustain autoantibody (auto-Ab) producing cells have not been definitively identified. Using BAFF-RFP reporter mice, we identified major changes in BAFF-producing cells in two mouse spontaneous lupus models (*Tlr7* Tg mice and *Sle1*), and in a pristane-induced lupus (PIL) model.

**Results:**

First, we confirmed that similar to their wildtype *Tlr7* Tg and *Sle1* mice counterparts, BAFF-RFP *Tlr7* Tg mice and BAFF-RFP *Sle1* mice had increased BAFF serum levels, which correlated with increases in plasma cells and auto-Ab production. Next, using the RFP reporter, we defined which cells had dysregulated BAFF production. BAFF-producing neutrophils (Nphs), monocytes (MOs), cDCs, T cells and B cells were all expanded in the spleens of BAFF-RFP *Tlr7* Tg mice and BAFF-RFP *Sle1* mice compared to controls. Furthermore, Ly6C^hi^ inflammatory MOs and T cells had significantly increased BAFF expression per cell in both spontaneous lupus models, while CD8^-^ DCs up-regulated BAFF expression only in the *Tlr7* Tg mice. Similarly, pristane injection of BAFF-RFP mice induced increases in serum BAFF levels, auto-Abs, and the expansion of BAFF-producing Nphs, MOs, and DCs in both the spleen and peritoneal cavity. BAFF expression in MOs and DCs, in contrast to BAFF from Nphs, was required to maintain homeostatic and pristane-induced systemic BAFF levels and to sustain mature B cell pools in spleens and BMs. Although acting through different mechanisms, Nph, MO and DC sources of BAFF were each required for the development of auto-Abs in PIL mice.

**Conclusions:**

Our findings underscore the importance of considering the relative roles of specific myeloid BAFF sources and B cell niches when developing treatments for SLE and other BAFF-associated autoimmune diseases.

## Introduction

B-cell-activating factor (BAFF) (also known as BLyS or Tnfsf13b) is a member of the TNF family of ligands that plays an essential role for mature B cells to develop, survive and respond to Ag (1–4). BAFF binds to three different TNF receptor family members: BAFFR, TACI and BCMA; signaling through these receptors functions as a key survival factor for maintaining mature B cell homeostasis (5–7). *Baff* ^-/-^ and *Baffr* ^-/-^ mice develop few or no mature B cells due to a block in B cell development from the newly formed transitional 1 (T1) to the T2 B cell stage onwards (5–7). We and others have shown that BAFF plays a key protective role against infectious diseases, including West Nile virus, HIV and *Salmonella typhimurium* (8–12).

On the other hand, BAFF overexpression contributes to the pathogenesis of a number of autoimmune diseases, including IgA nephropathy, type I diabetes, systemic sclerosis, Sjögren’s syndrome, rheumatoid arthritis, and systemic lupus erythematosus (SLE) (13–19). Mice overexpressing BAFF develop an SLE-like disease, and blocking BAFF receptors in these animals inhibits the development of kidney disease and prolongs survival (20, 21). Furthermore, mouse strains that develop spontaneous SLE have increased serum BAFF levels (14, 15, 20). Elevated serum BAFF levels and increased BAFF gene expression in peripheral blood cells are associated with disease activity in SLE patients (22–24). More recently, a genotype carrying a variant of the gene encoding BAFF was associated with increased disease severity in SLE (25).

The anti-BAFF Ab, belimumab, was the first BAFF-blocking drug approved for the treatment of SLE (15, 26–30). Other BAFF-targeting agents and belimumab in combination with other biologics are being assessed as treatments for lupus patients (29). The heterogeneity in clinical responses to belimumab and other BAFF-blocking drugs underscores the need to understand more fully the complexity of the BAFF system (3, 27, 28). While the downstream effects of blocking BAFF receptors have been examined extensively, the upstream regulation of BAFF production and how it affects the development of autoimmunity *in vivo* has not been investigated in detail.

In this study, we used BAFF-red fluorescent protein (RFP) reporter (BAFF-RFP) mice and BAFF conditional knockout (cKO) lines we previously developed (10) to identify BAFF sources implicated in the development of auto-Abs in lupus. We crossed BAFF-RFP reporter mice with two spontaneous mouse lupus models: *Tlr7* Tg mice (31–33) and *Sle1* mice (34, 35). Mice overexpressing TLR7 develop pathogenic auto-Abs and an SLE-like disease (33). *Tlr7* Tg mice also exhibit peripheral myeloid expansion, dependent on Type I IFN (IFN-I) signaling, a major BAFF inducer (10, 36, 37). *Sle1* (B6.NZMc1) mice contain an SLE-susceptibility locus that mediates the loss of tolerance to nuclear Ags and generates a robust, spontaneous anti-chromatin auto-Ab response that peaks at 7-9 mo of age (35, 38). In addition, we used a pristane-induced lupus (PIL) model (39) as an inducible lupus system readily suitable for studies using our BAFF cKO lines. The hydrocarbon oil pristane induces an SLE-like renal disease, ectopic lymphoid tissues and auto-Abs by 6-8 mo p.i (39). In the PIL model, similarly to *Tlr7* Tg mice, the SLE-like autoimmune phenotype is dependent on robust TLR7-mediated IFN-I production by inflammatory MOs (40, 41). In all three models, BAFF-RFP *Tlr7* Tg mice, BAFF-RFP *Sle1* mice, and pristane-treated BAFF-RFP mice, we observed increased BAFF serum levels, which correlated with increases in plasma cells (PCs) and auto-Ab production. BAFF-producing DCs, MOs and Nphs each expanded upon induction of autoimmunity, and each of these cellular sources of BAFF was required for the optimal induction of auto-Abs. However, DCs, MOs and Nphs regulated B cells and auto-Ab production through different mechanisms. DCs and MOs, but not Nphs, were required for the systemic production of BAFF and the maintenance of mature B cells in pristane-induce lupus. Our findings highlight how distinct BAFF sources may play different roles in the development of autoreactive B cells in lupus. The identification of specific BAFF-producing cells and niches implicated in SLE autoimmunity may be useful for designing new effective B cell targeted therapies.

## Materials and methods

### Mice

57BL/6J (WT) mice were purchased from Jackson Labs (Bar Harbor, ME). BAFF-RFP mice and *Baff^fl/fl^* mice (on the B6 background) were described previously (10). Briefly, BAFF-RFP reporter mice were generated by replacing a *Tnfsf13b/Baff* allele with a targeting construct expressing IRES-RFP. The *Tnfsf13b/Baff* is functionally knocked-out where the endogenous reporter expresses the RFP protein translated under the control of an IRES site. Thus, the BAFF-RFP signal is a measurement of BAFF expression and correlates with both *Baff* mRNA and BAFF protein expression (10). These heterozygous BAFF-RFP^+/-^ (BAFF-RFP) mice express the RFP protein on one *Baff* allele and the WT *Baff* on the other. *Tlr7.1* Tg mice and *Sle1* mice (31–36) (provided by KBE). *zDC^Cre^*, *Cx3cr1^Cre^* and *Mrp8^Cre^* mice were purchased from Jackson Labs (Bar Harbor, ME, USA). BAFF cKO mice where *Baff* is selectively deleted in either Nphs (*Baff^fl/fl^ Mrp8^Cre^*), cDCs (*Baff^fl/fl^ zDC^Cre^*) or MOs (*Baff^fl/fl^ Cx3cr1^Cre^*) were described previously (10). Briefly, a ~50% BAFF mRNA reduction was restricted to cDCs in *Baff^fl/fl^ zDC^Cre^*, and an ~85% reduction of BAFF mRNA was restricted to CX3CR1^hi^ MOs in *Baff^fl/fl^ Cx3cr1^Cre^*. In *Baff^fl/fl^ Mrp8^Cre^* BAFF mRNA was selectively, but not specifically reduced in Nphs by 85-90%, as some reduction was observed in MOs too. However, our previous studies do not show any overlap in the phenotype of *Baff^fl/fl^ Mrp8^Cre^* vs. *Baff^fl/fl^ Cx3cr1^Cre^* mice (10, 11). All mice were age- and sex-matched for experiments and used at 8-12 wks of age. Mice were housed in a specific pathogen free environment; all procedures were approved by the University of Washington Institutional Animal Care and Use Committee.

### Spontaneous lupus models

To generate BAFF-RFP expressing spontaneous lupus models, BAFF-RFP mice were crossed with *Tlr7.1* Tg mice or *Sle1* mice. Blood samples, spleens and bone marrows (BMs) were obtained from 6-8 months (mo) old BAFF-RFP *Tlr7.1* mice and *Tlr7.1* mice, and 7-9 mo BAFF-RFP *Sle1* mice and *Sle1* mice. WT mice and heterozygous BAFF-RFP controls were also included in the experiments.

### Pristane induced lupus

To induce a lupus-like disease, 0.5 ml or 0.4 ml of pristane (2,6,10,14-tetramethylpentadecane, TMPD) (Sigma-Aldrich, St. Louis, MO, USA) was administered by i.p. injection into 2-3 mo old mice (40). Controls mice were left untreated (naïve). Blood samples were obtained before pristane treatment and after treatment at day 7, day 14, day 28 and monthly thereafter. For short-term experiments with BAFF-RFP mice, spleens and peritoneal exudate cells (PECs) were harvested 2 wks after treatment. For long-term experiments with BAFF cKO mice, spleens and BMs were harvested 6 mo after pristane injections; untreated (naïve) and pristane treated mice were 8-9 mo old at the time of tissue harvest. Spleens and BMs from 2-3 mo old BAFF cKO mice and *Baff ^fl/fl^* mice were also harvested as controls at the time of injection.

### Tissue harvest and cell isolation

Spleens, BM cells and PECs were harvested and cell suspension obtained as described previously (9, 10, 42). Briefly, spleens were removed and dissociated by enzymatic digestion at 37°CC with liberase TL and Dnase I (Roche, Indianapolis, IN, USA), followed by mincing the tissue between the ends of two frosted microscope glass slides to obtain a single cell suspension. BM cells were isolated by cutting one end of the femur and flushing BM cells out of the bone by centrifugation. After erythrocytes were lysed, splenocytes, BM cells and PECs from peritoneal lavage were filtered and processed for staining for flow cytometry.

### Flow cytometry

Single cell suspensions obtained from spleens, BMs and PECs were incubated with Aqua Live-Dead fixable viability dye (Molecular Probes, Life Technologies, Waltham, MA, USA) in the absence of FBS, to discriminate dead cells. Cells were then blocked using an anti-F_C_ receptor Ab (anti-CD16/CD32) (2.4G2) (BioLegend, (San Diego, CA, USA) and stained for surface markers and then fixed in 1-2% paraformaldehyde. Cells were stained with mAbs conjugated to FITC, allophycocyanin, eFluor450, allophycocyanin-eFluor780, PerCPCy5.5, PE-Cy7, AlexaFluor647, BUV395, BV605, BV421, BV711, BV650 and BUV395. For the analyses of splenic, PEC- and BM cell-subsets, nine- to twelve-colors flow cytometry was performed using combinations of mAbs against: CD19 (1D3), CD11b (M1/70), IgM (II/41), CD5 (53-7.3), and CD11c (N418) from eBioscience (San Diego, CA, USA); B220 (RA3-6B2), CD93 (AA4.1), CD38 (90), GL7 (GL7), CD138 (281-2) and Ly6C (AL-21) from BD Horizon/Biosciences (San Jose, CA, USA); CD19 (1D3), B220 (RA3-6B2), CD3 (17A2), NK1.1 (PK136), CD8α (53-6.7), Ly6G (1A8), SiglecH (440c), CD21/35 (7E9), CD23 (B3B4), IgD (11-26c.2a), CD93 (AA4.1) and CD24 (M1/69) from BioLegend (San Diego, CA, USA). The BAFF-RFP signal was detected in the PE channel. Cells were processed with an LSRII flow cytometer (Becton Dickinson, Franklin Lakes, NJ, USA) using a FACSDiva software and data were analyzed using FlowJo (v.10, Tree Star). The gating strategy used for splenic, PEC and BM myeloid cell populations and T cells was described previously (9, 10, 42). For splenic myeloid cells, B cells (CD19^+^CD3^-^) and T cells (CD19^-^CD3^+^) were gated out; non-B cells and non-T cells (CD19^-^ CD3^-^ gate) populations were defined as follows: Nphs, CD11b^hi^Ly6G^hi^Ly6C^int^SSC^int-^NK1.1^-^; MOs, CD11b^hi^SSC^-^Ly6G^-^NK1.1^-^; Ly6C^lo^ MOs, CD11b^hi^Ly6C^lo^CD11c^-^SSC^-^Ly6G^-^NK1.1^-^; Ly6C^hi^ MOs, CD11b^hi^Ly6C^hi^CD11c^-^SSC^-^Ly6G^-^NK1.1^-^; Ly6C^hi^ DCs, CD11b^hi^Ly6C^hi^CD11c^hi^ SSC^-^Ly6G^-^NK1.1^-^; CD8^+^ cDCs, CD11c^hi^CD8^+^B220^-^Ly6G^-^NK1.1^-^; CD8^-^ cDCs, CD11c^hi^CD8^-^B220^-^Ly6G^-^NK1.1^-^. In BM, myeloid cells were defined in B220^-^CD19^-^NK1.1^-^SiglecH^-^CD11b^+^ gate as follows: Nphs, Ly6G^hi^Ly6C^int^SSC^int-^; Ly6C^hi^ MOs, CD11c^-^CD115^+^CX3CR1^+^CCR2^hi^ MHCII^-^Ly6G^-^SSC^-^; preDCs, CD11c^+^Ly6C^-^CD115^+^CX3CR1^hi^CCR2^+^MHCII^+^Ly6G^-^SSC^-^. BM T cells were defined as CD3^+^ CD19^-^ in the NK1.1^-^SiglecH^-^CD11b^-^ gate. In PECs Nphs were defined as CD19^-^B220^-^CD11b^hi^Ly6G^hi^Ly6C^int^SSC^int-^; other myeloid cells in the gate CD19^-^B220^-^CD11b^+^Ly6G^lo-^SSC^lo^ were defined as follows: Ly6C^hi^ MO, Ly6C^hi^ CD11c^-^; Ly6C^hi^ Mph/DC, Ly6C^hi^ CD11c^+^; Mph, Ly6C^int^ CD11c^lo^; DC, Ly6C^lo^ CD11c^hi^.

Splenic B cells subsets were defined as described previously (9, 10, 43). After gating out debris, doublets and dead cells, B cell subsets in the CD19^+^ B220^+^ gate were defined as follows: Follicular (FO) B cells, CD24^mid^CD21/35^mid^CD93^-^CD23^-^; Marginal zone (MZ) B cells, CD24^hi^CD21/35^hi^CD93^-^CD23^-^; MZ B cell precursors (MZP), CD24^hi^CD21/35^hi^CD93^lo^CD23^+^; Newly-formed transitional 2 (T2) B cells, CD24^hi^CD21/35^int/hi^CD93^+^CD23^+^; Newly-formed transitional 1 (T1) B cells CD24^hi^CD21/35^lo^CD93^+^CD23^-^. Germinal center (GC) B cells were defined as CD19^+^ B220^+^CD38^-^GL7^+^. BM B cell precursors and PCs were gated as previously described (10). NK cells, pDCs and CD11b^+^ cells were gated out and in the NK1.1^-^SiglecH^-^CD11b^-^ gate we defined PreProB cells as B220^-^CD43^+^CD19^-^; ProB cells as B220^lo^CD43^+^CD19^+^; PreB cells as B220^+^CD43^-^CD19^+^IgM^-^IgD^-^; newly formed B cells (NFB) as B220^+^CD43^-^CD19^+^IgM^+^IgD^-^; mature B cells (Mature B) as B220^+^CD43^-^CD19^+^IgM^+^IgD^+^; PEC B cells were defined as B220^+^CD19^+^ (CD5^-^CD23^+^IgM^lo^IgD^hi^) B cells and B1 B cells B220^lo-^CD19^hi^CD23^-^IgM^hi^IgD^lo^, subsequently subdivided in CD5^+^ B1a B cells and CD5^-^ B1b cells. Splenic PCs, PEC PCs and long-lived PCs in the BM were defined as B220^lo^CD138^+^.

### Auto-Ab and BAFF ELISAs

Sera were isolated from blood, collected *via* the retro-orbital route. Serum levels of BAFF were determined using the Mouse BAFF/BLyS/TNFSF13B DuoSet ELISA kit (R&D systems, Inc., Minneapolis, MN) according to the manufacturer’s instructions. For specific auto-Ab ELISAs (44), 96 well Immuno plates (Nunc) were coated with: Sm/RNP (5μg/ml; Arotec Diagnostic Limited ATR01-10); yeast RNA (5μg/ml; Sigma R6750); calf thymus dsDNA (25μg/ml; Sigma-Aldrich D4522) or calf thymus histone (10μg/ml; Sigma-Aldrich H9250) diluted in 1X PBS and incubated overnight at 4°C. Prior to coating with yeast RNA and calf thymus histone, the plates were coated with Poly L Lysine (25μg/ml; Sigma P6516) for 2 h at RT or overnight at 4°C. Plates were then blocked for 2 h at RT with 1% BSA/PBS prior to addition of diluted serum for 2 h at RT. For ssRNA ELISA plates were blocked with 5% Goat serum/PBS for 2 h at RT and diluted serum were incubated overnight at 4°C. Specific Ab were detected using goat anti-mouse IgG Ab coupled with HRP (Southern Biotech, Birmingham, AL) and allowed to incubate for 2 h at RT. HRP activity was visualized using tetramethylbenzidine peroxidase (Bio-Rad Laboratories, Hercules, CA). Absorbance for all ELISA assays were measured at 450 nm with a Synergy plate reader (BioTek). For the standard curve, goat anti-mouse IgG (Southern Biotech, Birmingham, AL) was used for coating, and serial dilutions of recombinant IgG (Southern Biotech, Birmingham, AL) were used to calculate the absolute quantities of anti-SmRNP IgG and anti-ssRNA IgG. Serial dilutions of sera were used to determine the relative amount of anti-dsDNA and anti-Histone in different groups, measured by the OD at 450nm.

### Statistical analyses

Timeline data were analyzed using a 2-way ANOVA with Tukey’s multiple comparison test. Analyses between more than two groups were performed using 1-way ANOVA with Holm-Sidak method for multiple comparisons. Analyses between two groups were performed using unpaired Student t test or Mann Whitney test. GraphPad Prism was used for statistical analyses. Differences of *p*<0.05 were considered significant.

## Results

### Induction of BAFF, autoantibodies and plasma cells in BAFF-RFP *Tlr7.1* Tg mice and BAFF-RFP *Sle1 mice*

**Figure 1:**
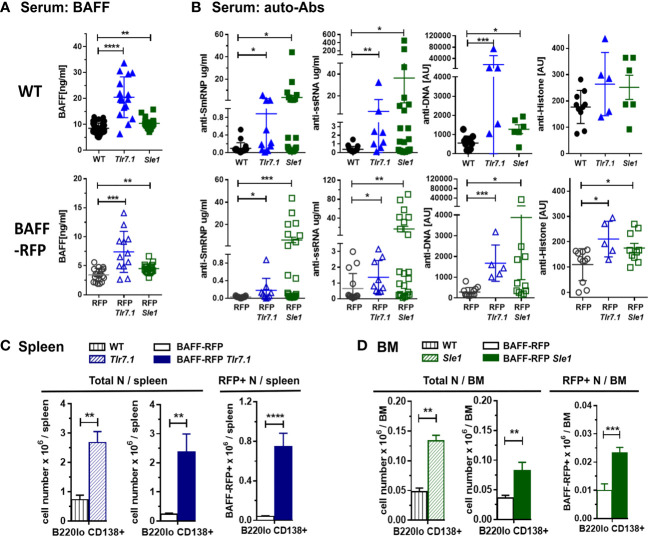
Induction of BAFF, auto-antibodies and PCs in BAFF-RFP *Tlr7.1* mice and BAFF-RFP *Sle1* mice. BAFF-RFP (BAFF-RFP^+/-^) mice were crossed with *Tlr7.1* mice and *Sle1* mice, 6-8 mo old *Tlr7.1* genotypes and 7-9 mo old *Sle1* genotypes were analyzed **(A**, **B)** Serum from WT, *Tlr7.1* and *Sle1* mice (*upper panels*) and BAFF-RFP, BAFF-RFP *Tlr7.1* and BAFF-RFP *Sle1* mice (*lower panels*) were collected and analyzed by ELISA for BAFF titers **(A)** and anti-SmRNP, anti-ssRNA, anti-DNA and anti-Histone auto-Abs **(B)**. **(A)** graphs summarize BAFF data from more than three independent experiments (WT, *Tlr7.1* and *Sle1* mice: N=16-43; BAFF-RFP, BAFF-RFP *Tlr7.1* and BAFF-RFP *Sle1*: N=12-19). **(B)** graphs summarize auto-Ab data from more than two independent experiments (WT, *Tlr7.1* and *Sle1* mice: N=5-20; BAFF-RFP, BAFF-RFP *Tlr7.1* and BAFF-RFP *Sle1*: N=5-22). **(C**, **D)**, Spleens from BAFF-RFP *Tlr7.1*, BAFF-RFP, *Tlr7.1* and WT mice **(C)** and BM from BAFF-RFP *Sle1*, BAFF-RFP, *Sle1* and WT mice **(D)** were harvested and analyzed by flow cytometry for B220^lo^ CD138^+^ PC numbers. Total PC numbers in **(C, D)** *left panels*, and BAFF-RFP^+^ PC numbers in **(C**, **D)** *right panels*, are shown as bar graphs (mean ± SEM) and summarize data from three independent experiments (WT, *Tlr7.1* and *Sle1* mice: N=7-9; BAFF-RFP, BAFF-RFP *Tlr7.1* and BAFF-RFP *Sle1*: N=9-13). Statistics were performed using one-way ANOVA with Holm-Sidak method for multiple comparisons tests **(A, B)**, or two-tailed unpaired Student t test **(C, D)**; *p<0.05, **p<0.01, ***p<0.001, ****p<0.0001.

To investigate BAFF regulation and BAFF-producing cells in lupus prone mice, we crossed our BAFF-RFP reporter mice with *Tlr7.1* Tg mice and *Sle1* mice. Heterozygous BAFF-RFP^+/-^ (BAFF-RFP) mice express lower BAFF levels, as one *Baff* allele expresses the RFP protein as a measurement of BAFF expression and the other allele expresses wild-type (WT) *Baff* (10, 11). However, BAFF-RFP still retain a reduced population of mature B cells, and thus can be used for functional studies of B cells and Ab responses (10, 11). We first assessed how either the *Tlr7.1* Tg or the *Sle1* background affected serum BAFF levels. Both 6-8 mo old *Tlr7.1* Tg mice and the 7-9 mo old *Sle1* mice had increased serum BAFF levels compared to age-matched WT control B6 mice, although the *Tlr7.1* Tg mice had more pronounced increases in serum BAFF compared to the *Sle1* mice (**Figure 1A**, *upper panel*). Similarly, BAFF levels in the sera of BAFF-RFP *Tlr7.1* Tg mice and BAFF-RFP *Sle1* mice were significantly up-regulated compared to BAFF-RFP control mice (**Figure 1A**, *lower panel*). As expected, since the heterozygous BAFF-RFP lines express half the normal levels of BAFF protein, they had about half the BAFF serum levels of the parental lines (**Figure 1A**).

Next, we tested whether the *Tlr7.1* Tg or *Sle1* background was sufficient to induce an autoimmune phenotype in the BAFF-RFP mice, even in the context of reduced mature B cells. As expected, *Tlr7.1* Tg mice and *Sle1* control mice had elevated titers of anti-SmRNP, -ssRNA and -dsDNA IgG auto-Abs compared to WT mice (**Figure 1B**, *upper panels*). Similarly, BAFF-RFP *Tlr7.1* Tg mice and BAFF-RFP *Sle1* mice had substantially increased serum auto-Ab levels compared to BAFF-RFP controls (**Figure 1B**, *lower panels*). Surprisingly, the mean levels of serum IgG auto-Abs in BAFF-RFP *Sle1* mice were comparable to those in *Sle1* mice. Furthermore, the fold increase in anti -SmRNP and -dsDNA IgG auto-Abs in BAFF-RFP *Sle1* mice compared to BAFF-RFP mice was greater than in *Sle1* mice compared to WT controls (SmRNP: 226 -fold in RFP *Sle1/*RFP in vs 37 -fold in *Sle1*/WT; dsDNA: 13.4-fold in RFP *Sle1/*RFP vs 2.3 -fold in *Sle1*/WT). Anti-histone IgG Abs were also up-regulated to a similar extent in BAFF-RFP *Tlr7.1* Tg mice and *Tlr7.1* Tg mice (**Figure 1B**).

Consistent with the increased auto-Ab levels, spleens of BAFF-RFP *Tlr7.1* Tg mice had increased PC numbers compared to BAFF-RFP mice and were comparable to those of *Tlr7.1* Tg mice (**Figure 1C** and **Supplementary Figure S1A**). PCs were not upregulated in the BM of either *Tlr7.1* Tg mice or BAFF-RFP *Tlr7.1* Tg mice (data not shown). *Sle1* mice had expanded levels of PCs in the BM (**Figure 1D** and **Supplementary Figure S1B**), and to a lesser extent, in the spleen (**Supplementary Figure S1C**). We previously showed that PCs are also BAFF producers (10). BAFF-RFP PCs were also substantially expanded in the spleens of BAFF-RFP *Tlr7.1* Tg mice (**Figure 1C**, *right panel*) and in the BM of BAFF-RFP *Sle1* mice (**Figure 1D**, *right panel*). In summary, BAFF-RFP mice with either the *Tlr7.1* Tg or *Sle1* lupus genotype developed an autoimmune phenotype comparable to their parental line including higher systemic BAFF levels, expanded PCs in spleens and BMs, and increased anti-chromatin auto-Abs.

### Regulation of BAFF-RFP expression in splenic and BM myeloid and lymphoid cells from BAFF-RFP *Tlr7.1* and *Sle1* mice

**Figure 2:**
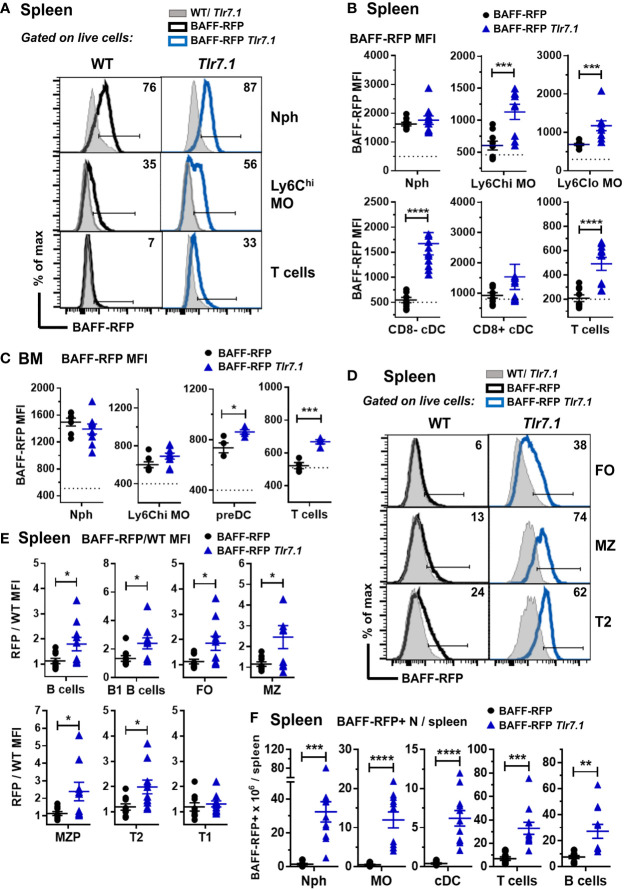
BAFF-RFP expression is upregulated in splenic and BM myeloid cells and in splenic T and B cells from BAFF-RFP *Tlr7.1* mice. Spleens **(A, B, D-F)** and BM **(C)** from 6-8 mo old BAFF-RFP *Tlr7.1*, BAFF-RFP, *Tlr7.1* and WT mice were harvested, and cell populations were analyzed by flow cytometry. For the gating strategy used for myeloid cells, B and T cells see *Methods*. **(A-C**, **F)**, Nph, Ly6c^hi^ and Ly6C^lo^ MOs, CD8^-^ and CD8^+^ cDCs, DC precursors (preDC), T cells. **(D–F)**, total CD19^+^ B220^+^ B cells, B1 B cells, FO B cells, MZ B cells, MZ precursors (MZP), T2 and T1 B cells. Data shown are representative histograms **(A, D)**, or dot plot graphs showing mean ± SEM of RFP MFI (Median Fluorescence Intensity) **(B, C, E)**, and numbers of BAFF-RFP^+^ cell populations per spleen **(F)**, from three independent experiments (WT and *Tlr7.1* mice: N=7-8; BAFF-RFP and BAFF-RFP *Tlr7.1*: N=9-11). In A and D numbers indicate % of BAFF-RFP^+^ cells. In B and C dotted lines show RFP MFI background in WT control. In E, data are shown as ratio of RFP/WT MFI due to the increased RFP signal background in *Tlr7.1* vs WT B cells. Statistics were performed by one-way ANOVA with Holm-Sidak method for multiple comparisons; *p<0.05, **p<0.01, ***p<0.001, ****p<0.0001.

Next, we investigated how a *Tlr7* or *Sle1* background affected BAFF expression in myeloid and lymphoid subsets. Compared to controls, BAFF-RFP *Tlr7.1* mice had increased BAFF expression in all splenic MO subsets and in CD8^-^ DCs (**Figures 2A, B**) as well as in BM preDCs (**Figure 2C**). Interestingly, TLR7 overexpression did not affect BAFF expression in either splenic or BM Nphs, which constitutively express high BAFF levels (**Figures 2A–C**). Surprisingly, TLR7 overexpression also upregulated BAFF in splenic and BM T cells (**Figures 2A–C**) as well as in several splenic B cell subsets including B1 B cells (**Figures 2D, E**). The mature FO and MZ B cells and T2 B cells from BAFF-RFP *Tlr7.1* mice had increased BAFF expression, while T1 B cells were not affected (**Figure 2E**). TLR7 overexpression, in addition to up-regulating BAFF expression on a per cell basis, induced the expansion of several BAFF-producing myeloid and lymphoid splenic cell populations (**Figure 2F**).

**Figure 3:**
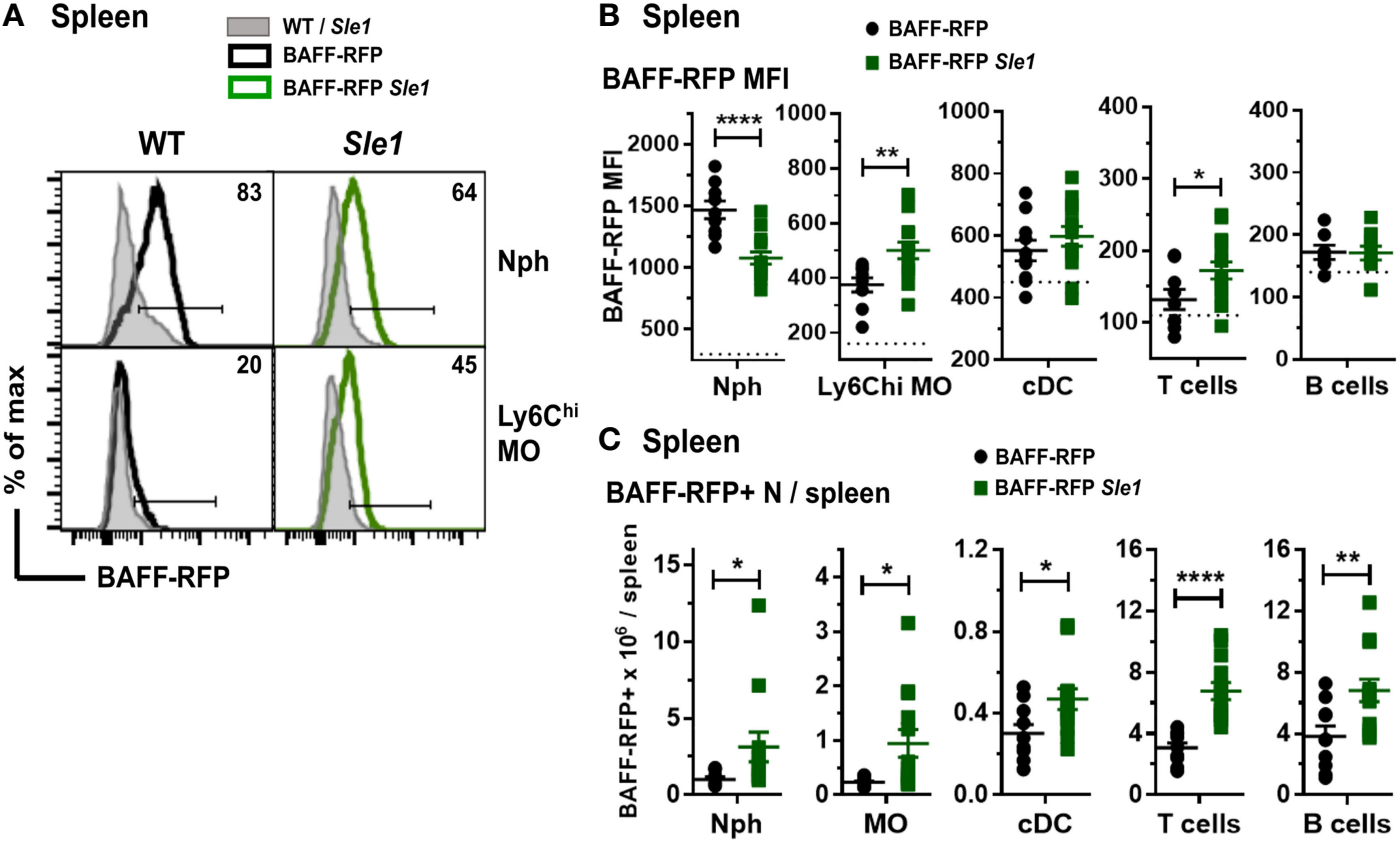
Changes in BAFF-RFP expression in splenic myeloid cells from BAFF-RFP *Sle1* mice. Spleens from 7-9 mo old BAFF-RFP *Sle1*, BAFF-RFP, *Sle1*, WT mice were harvested, and cell populations were analyzed by flow cytometry. For gating strategy of splenic myeloid cells, B and T cells see *Methods*. Data shown are representative histograms **(A)**, or dot plot graphs showing mean ± SEM of RFP MFI **(B)**, and numbers of BAFF-RFP^+^ cell populations per spleen **(C)**, from three independent experiments (WT and *Sle1* mice: N=9; BAFF-RFP and BAFF-RFP *Sle1*: N=9-13). In **A** numbers indicate % of BAFF-RFP^+^ cells. In **(B)** dotted lines show RFP MFI background in WT control. Statistics were performed by one-way ANOVA with Holm-Sidak method for multiple comparisons; *p<0.05, **p<0.01, ****p<0.0001.

Myeloid and lymphoid cells were not as profoundly expanded in the *Sle1* mice as in the *Tlr7.1 Tg* mice (45). However, similar to the BAFF-RFP *Tlr7.1* mice, BAFF was up-regulated in splenic inflammatory Ly6C^hi^ MOs and to a lower extent in T cells from BAFF-RFP *Sle1* mice (**Figures 3A, B**). In contrast to the TLR7 Tg mice, splenic Nphs from BAFF-RFP *Sle1* mice had lower BAFF expression per cell than Nphs from BAFF-RFP controls (**Figures 3A, B**), possibly due to Nph activation. Indeed, we and others have shown that upon activation, Nphs not only release BAFF from the intracellular stores but also down-regulate BAFF expression (11). cDC- and B cell-BAFF expression per cell did not change in the spleens of *Sle1* autoimmune mice (**Figure 3B**). However, similar to TLR7 overexpression, *Sle1* induced the expansion of several BAFF-producing lymphoid and myeloid cell populations, including Nphs (**Figure 3C**). Thus, in both *Tlr7.1* Tg and *Sle1* spontaneous SLE models, increased systemic BAFF levels and the development of autoimmunity were associated with a significant expansion of all the major BAFF-producing myeloid cell populations: Nphs, MOs, and cDCs.

### Increased serum BAFF and expansion of BAFF-RFP^+^ myeloid cells in the spleen and peritoneal cavity of pristane treated lupus-prone mice

**Figure 4:**
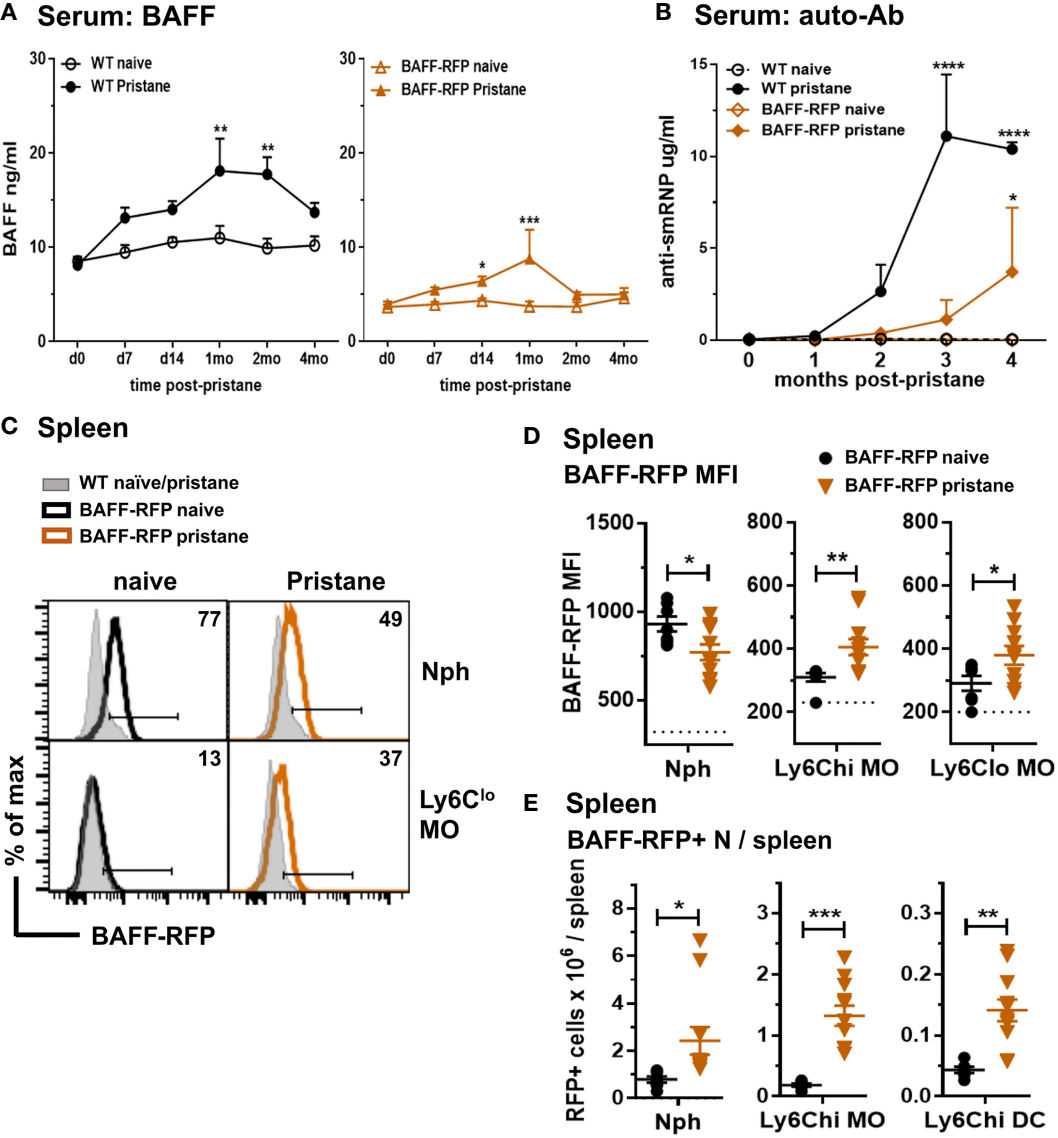
Increase in serum BAFF and auto-Abs and expansion of BAFF-RFP^+^ myeloid cells in spleen after pristane treatment. WT and BAFF-RFP mice were injected or not with 500µl pristane i.p. Serum was collected at the indicated time points, BAFF **(A)** and auto-Abs **(B)** were measured by ELISA. **(A)**, BAFF titers in naïve and pristane-treated WT mice (*left panel*) and BAFF-RFP mice (*right panel*). **(B)**, Anti-SmRNP IgG in pristane-treated WT and BAFF-RFP mice, naïve mice are shown as controls. In **(A**, **B)** graphs show representative data from one of two independent experiments with at least three mice per group. Statistics were performed using 2-way ANOVA with Tukey’s multiple comparison test; **p<0.01, ****p<0.0001. In **(A**, **B)** statistics shown are the comparison between pristane-treated and naïve mice in each group (WT pristane vs WT naïve and BAFF-RFP Pristane vs. BAFF-RFP naïve). In **(C–E)**, two weeks after pristane injection spleens were harvested, and splenic cell populations were analyzed by flow cytometry. For gating strategy see *Methods*. Data are shown as representative BAFF-RFP histograms **(C)**, or dot plots graphs showing mean ± SEM of RFP MFI **(D)**, and numbers of BAFF-RFP^+^ cell populations per spleen **(E)** summarizing data from two independent experiments (naïve and pristane treated WT mice: N=4-6; naïve and pristane treated BAFF-RFP mice: N=7-11). In **(C)** numbers indicate % of BAFF-RFP^+^ cells. In **(D)** dotted lines show RFP MFI background in WT control. Statistics were performed by one-way ANOVA with Holm-Sidak method for multiple comparisons; *p<0.05, **p<0.01, ***p<0.001.

**Figure 5:**
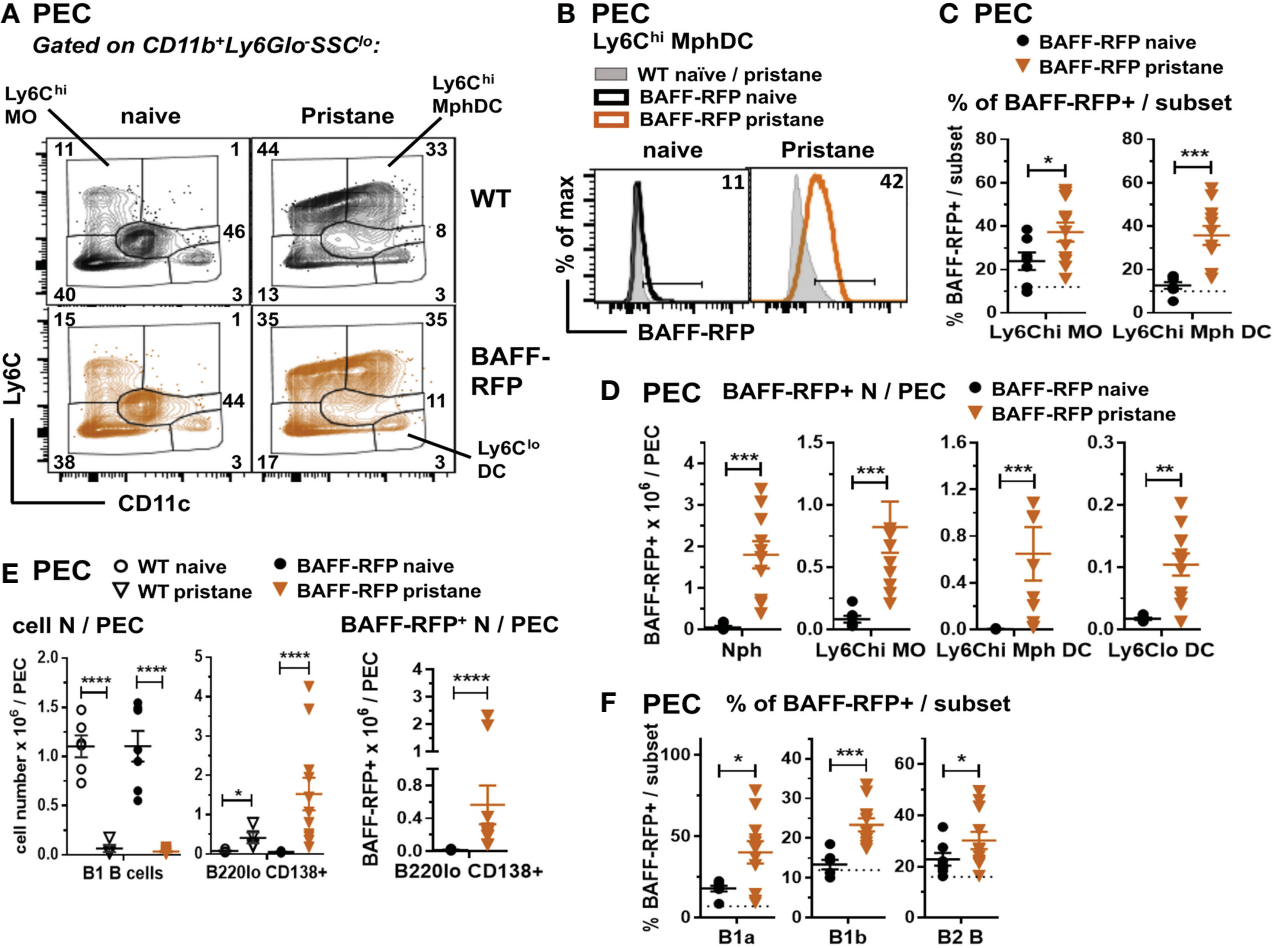
Expansion of BAFF-RFP^+^ Nphs and inflammatory MO/Mph/DCs and PCs in the peritoneal cavity after pristane injection. WT and BAFF-RFP mice were injected or not with 500µl pristane i.p.; two weeks later peritoneal cavity exudate cells (PEC) were harvested, and cell populations were analyzed by flow cytometry. For gating strategy of myeloid cells **(A–D)** and B cells **(E, F)** see *Methods*. Data are shown as representative dot plots **(A)**, BAFF-RFP histograms **(B)**, or dot plot graphs showing mean ± SEM of the % of BAFF-RFP^+^ cells per subset **(C, F)**, numbers of BAFF-RFP^+^ cell populations per spleen (**D**, **E** *left panel*), and total numbers cell subsets per spleen (**E** *left and central panel*) summarizing data from two independent experiments (naïve and pristane-treated WT mice: N=4-6; naïve and pristane-treated BAFF-RFP mice: N=7-11). In **(B)** numbers indicate % of BAFF-RFP^+^ cells. In **(C**, **F)** dotted lines show RFP background in WT control. Statistics were performed by one-way ANOVA with Holm-Sidak method for multiple comparisons; *p<0.05, **p<0.01, ***p<0.001, ****p<0.0001.

In order to explore the effects of depletion of BAFF in specific immune cell types, we used a pristane-induced mouse model of lupus (10, 11). As with *Tlr7*Tg mice, the SLE-like autoimmune phenotype of PIL mice is dependent on IFN-I production and expansion of inflammatory myeloid cells (39). Pristane treatment of WT B6 mice induced an increase in serum BAFF levels that peaked at 30 days post-injection (**Figure 4A**, *left panel*). As with WT mice, BAFF-RFP mice up-regulated serum BAFF that peaked and doubled 30 days after pristane injection (**Figure 4A**, *right panel*). Since the BAFF-RFP mice express only one wild-type BAFF allele, constitutive and induced serum BAFF levels were lower in these mice than in WT mice (**Figure 4A**). As expected from previous studies in B6 mice (46), WT mice had a modest up-regulation of anti-SmRNP by 3-4 mo post pristane injection (**Figure 4B**). In pristane-treated BAFF-RFP mice, auto-Ab responses were delayed and lower than in WT mice, but still detectable and significantly different from BAFF-RFP untreated mice (**Figure 4B**). As previously reported (38), pristane did not induce anti-DNA or anti-histone auto-Abs in the B6 strain (data not shown). Reeves et al. showed that pristane injection induces recruitment of Nphs and inflammatory MOs to the peritoneal cavity and spleen, peaking two weeks post-injection (41). At this time point, BAFF-RFP expression was up-regulated in splenic MO subsets, but decreased in Nphs from BAFF-RFP mice, similar to what we detected in BAFF-RFP *Sle1* mice (**Figures 4C, D**). Also, as in the spontaneous lupus models, BAFF^+^ Nphs and BAFF^+^ inflammatory MOs/DCs were expanded in the spleens of pristane-treated BAFF-RFP mice (**Figure 4E**). As expected, two weeks after pristane treatment Ly6C^hi^ inflammatory MO and mature CD11c^+^ Mph/DCs were also expanded in the peritoneal cavity of BAFF-RFP mice, as well as in WT mice (**Figure 5A**). These inflammatory MOs and Mph/DCs also up-regulated BAFF-RFP expression on a per cell basis (**Figures 5B, C**), and together with BAFF-RFP+ Nphs, were substantially increased in numbers after pristane injection (**Figure 5D**).

Together with the significant expansion of myeloid populations, peritoneal B2 and B1 B cells are substantially reduced by pristane treatment (41). Interestingly, although B1 B cells were reduced in the peritoneal cavity of either WT or BAFF-RFP mice, PC numbers and BAFF-RFP^+^ PCs were expanded after pristane injection (**Figure 5E**). Furthermore, the remaining B1a and B1b B cells, and to a lesser extent B2 B cells, normally expressing low BAFF levels, upregulated BAFF-RFP expression upon pristane injection (**Figure 5F**). Thus, our data suggest that in PIL, as well as in the spontaneous lupus models (**Figures 1C, D**; **2D–F**; **3C**), autocrine and paracrine BAFF from PCs and other B cell subsets could also contribute to sustain B cells.

In summary, similar to the spontaneous lupus models, PIL induced the expansion of splenic and peritoneal BAFF producing -Nphs and -inflammatory MOs/DCs and an increase in systemic BAFF levels, together with the expansion of PCs and induction of auto-Abs.

### BAFF from MOs and cDCs is required for homeostatic and pristane–induced systemic BAFF levels

**Figure 6:**
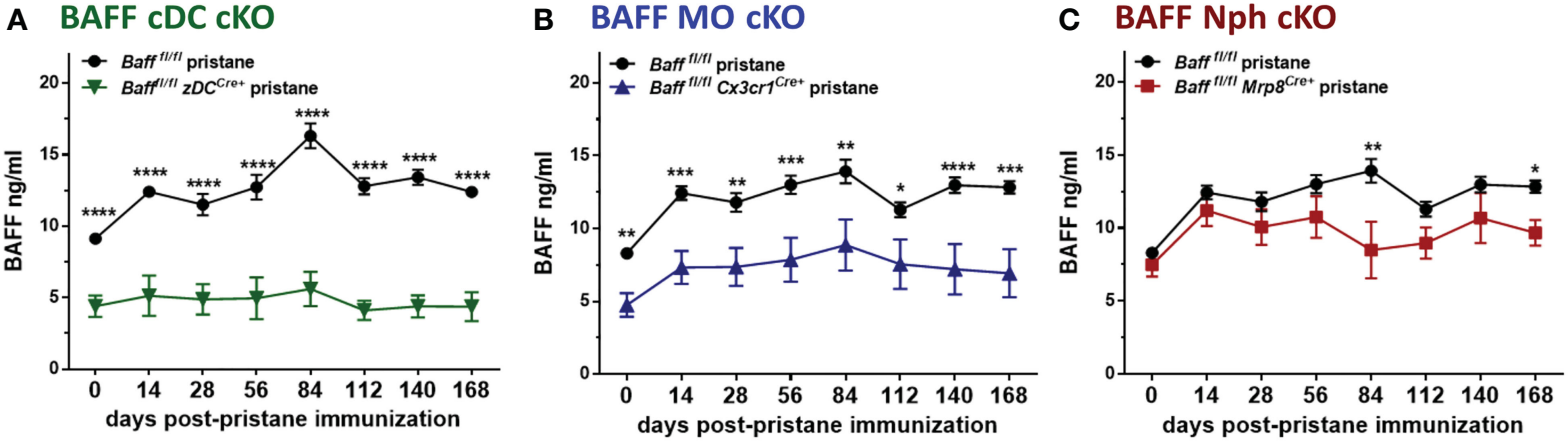
BAFF from MOs and cDCs is required to maintain homeostatic and pristane–induced systemic levels of BAFF. **(A-C),** *Baff^fl/fl^*, **(A)** *Baff^fl/fl^ zDC^Cre+^* mice (BAFF cDC cKO), **(B)** *Baff^fl/fl^ Cx3cr1^Cre+^* mice (BAFF MO cKO) and **(C)** *Baff^fl/fl^ Mrp8^Cre+^* mice (BAFF Nph cKO) were treated for 6 mo with 400µl pristane. At the indicated time points sera were collected and BAFF levels were measured by ELISA. Graphs summarize data from two to three independent experiments with three or more mice per group (*Baff^fl/fl^*, N=6-21; *Baff^fl/fl^ zDC^Cre+^*, N=6-14; *Baff^fl/fl^ Cx3cr1^Cre+^*, N=13-18; *Baff^fl/fl^ Mrp8^Cre+^*, N=9-10). Statistics were performed using 2-way ANOVA with Tukey’s multiple comparison test. *p<0.05, **p<0.01, ***p<0.001, ****p<0.0001.

Since myeloid cells have been implicated as the major producers of BAFF (10), and were expanded in spontaneous lupus models as well as in PIL, we asked whether deletion of BAFF in one of the major subpopulations of myeloid cells would influence BAFF levels and downstream effects in PIL. Surprisingly, already at the time of pristane injection (day 0) 2-3 mo old naïve *Baff^fl/fl^ zDC^Cre+^* (BAFF cDC cKO) mice and *Baff^fl/fl^ Cx3cr1^Cre+^* (BAFF MO cKO) mice, but not *Baff^fl/fl^ Mrp8^Cre+^* (BAFF Nph cKO) mice had lower BAFF levels in sera compared to naive *Baff ^fl/fl^* mice (**Figures 6A–C** and **Supplementary Figure S2A**). Over a period of 6 mo, only BAFF cDC cKO mice had significantly reduced BAFF levels compared to naive *Baff ^fl/fl^* mice (**Supplementary Figure S2A**). Furthermore, while pristane treatment of *Baff ^fl/fl^* control mice induced a sustained increase in serum BAFF levels, it did not up-regulate systemic BAFF levels in BAFF cDC cKO mice for up to 6 mo post-injection (**Figure 6A** and **Supplementary Figure S2B**). In mice lacking BAFF in MOs, serum BAFF slightly increased in the first few months post-pristane injection but was significantly lower than in *Baff ^fl/fl^* control mice over the course of the experiment (**Figure 6B** and **Supplementary Figure S2C**). In contrast, BAFF Nph cKO mice initially up-regulated systemic BAFF levels to a similar extent as *Baff ^fl/fl^* control mice, but at 3 and 6 mo post-pristane injection BAFF was significantly lower in these mice than in the controls (**Figure 6C** and **Supplementary Figure S2C**). Serum BAFF levels in *zDC^Cre+^*, *Cx3cr1^Cre+^* and *Mrp8^Cre+^* control mice were similar to *Baff ^fl/fl^* mice (**Supplementary Figure S2D**). Therefore, cDC-BAFF is required to sustain homeostatic levels of BAFF in the serum, while BAFF from either MOs or cDCs is required for pristane-induced systemic BAFF levels. Although Nphs are major BAFF producers (10), Nph-BAFF is not required to sustain homeostatic BAFF levels in the serum, but does contribute to the upregulation of serum BAFF at later stages of pristane-induced autoimmunity.

### BAFF from DCs and MOs skews MO differentiation toward a more inflammatory Ly6C^hi^ MO during long-term pristane treatment

**Figure 7:**
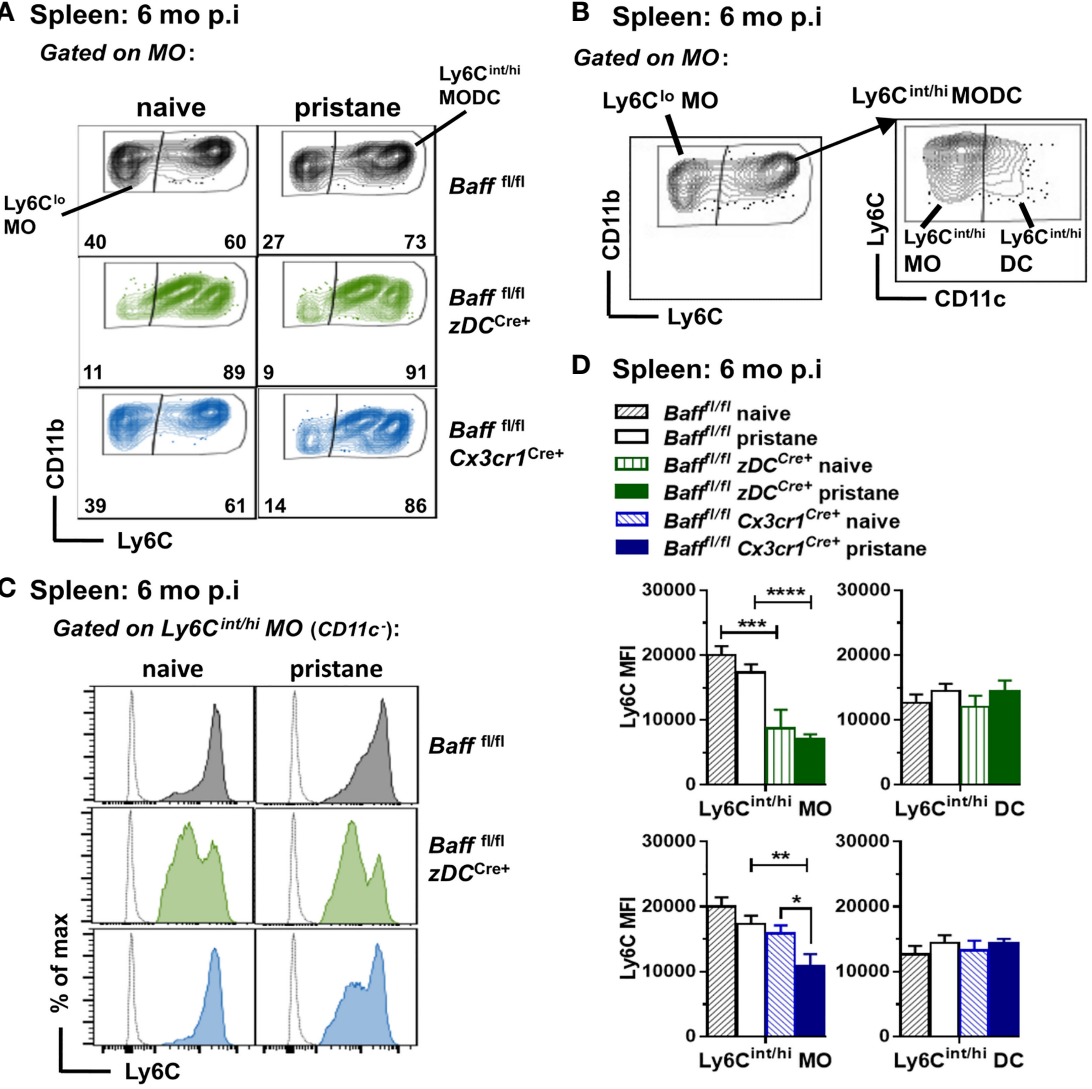
BAFF from DCs and MOs after pristane injection skews MO differentiation toward Ly6C^hi^ inflammatory MOs. *Baff^fl/fl^*, *Baff^fl/fl^ zDC^Cre+^* mice (BAFF cDC cKO) and *Baff^fl/fl^ Cx3cr1^Cre+^* mice (BAFF MO cKO) were injected or not with 400µl pristane; 6 mo later splenic MO subsets were analyzed by flow cytometry (see *Methods* for gating strategy). **(A)**, Representative dot plots of MO subsets in all the groups. **(B)**, Representative dot plots for MO subsets gating strategy to identify Ly6C^int/hi^ MO and MO-derived DCs. **(B),** *left panel* shows Ly6C^lo^ MO and Ly6C^int/hi^ MODC in the MO gate (CD19^-^ CD3^-^ NK1.1^-^ CD11b^hi^ Ly6G^-^ SSC-H^lo^); *right panel* shows Ly6C^in/hi^ MOs (CD11c^-^) and Ly6C^int/hi^ DCs (CD11c^+^) in the Ly6C^int/hi^ MODC population. **(C)**, shows representative Ly6C histograms of Ly6C^in/hi^ MOs (CD11c^-^). **(D)**, shows bar graphs of Ly6C MFI in Ly6C^in/hi^ MO and Ly6C^int/hi^ DC subsets, summarizing data from two to three independent experiments (*Baff^fl/fl^* naive, N=8; *Baff^fl/fl^* pristane, N=18; *Baff^fl/fl^ zDC^Cre+^* naive, N=3; *Baff^fl/fl^ zDC^Cre+^* pristane, N=10; *Baff^fl/fl^ Cx3cr1^Cre+^* naive, N=4; *Baff^fl/fl^ Cx3cr1^Cre+^* pristane, N=12). In **(A)** numbers indicate % of cells in Ly6C^lo^ MO and Ly6C^hint/hi^ MODC subsets. Statistics were performed by one-way ANOVA with Holm-Sidak method for multiple comparisons; *p<0.05, **p<0.01, ***p<0.001, ****p<0.0001.

We next analyzed whether selective depletion of BAFF from DCs, MOs or Nphs altered splenic myeloid and B cell populations. Unexpectedly, we found that BAFF depletion from cDCs or MOs, but not Nphs, affected the phenotype of inflammatory Ly6C^hi^ MO in the spleen (47–49). Specifically, splenic Ly6C^int/hi^ MOs from either 8-9 mo-old untreated or pristane-treated BAFF cDC cKO mice had lower Ly6C MFI levels compared to those from *Baff ^fl/fl^* mice (**Figures 7A–D**). The same phenotype was observed in mice lacking BAFF in MOs, but only after pristane injection (**Figures 7A–D**). In contrast to older mice, Ly6C^int/hi^ MOs from 2-3 mo old mice were not significantly different in BAFF cDC cKO mice, BAFF MO cKO mice and *Baff ^fl/fl^* mice (data not shown). No changes in Ly6C MFI occurred in Ly6C^int/hi^ DCs (CD11c^+^) from BAFF cDC cKO and BAFF MO cKO mice (**Figures 7B, D**), suggesting that lack of BAFF production by cDCs or MOs, skewed MO differentiation toward a Ly6C^int^ MO/Mph subset rather than MO-derived DCs. Thus, in an autoimmune setting, DC-BAFF and MO-BAFF were required to maintain high expression of Ly6C in inflammatory MOs.

### BAFF from MOs and cDCs is required to sustain mature B cell pools in spleen and BM in pristane-treated mice

**Figure 8:**
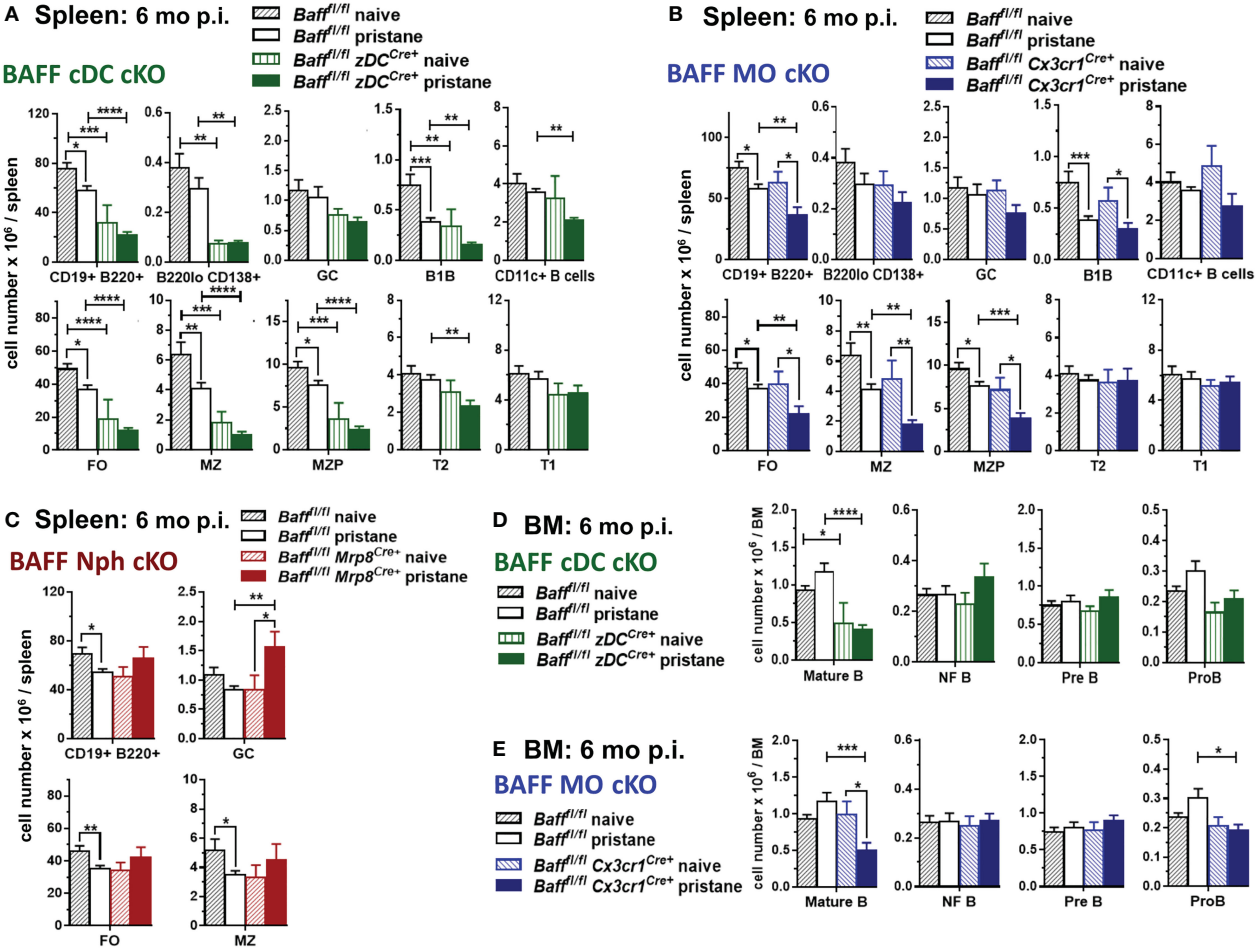
BAFF from cDCs and MOs is required for long-term maintenance of mature splenic and BM B cells after pristane treatment. *Baff^fl/fl^* mice, *Baff^fl/fl^ zDC^Cre+^* mice (BAFF cDC cKO) **(A, D)**, *Baff^fl/fl^ Cx3cr1^Cre+^* mice (BAFF MO cKO) **(B, E)** and *Baff^fl/fl^ Mrp8^Cre+^* mice (BAFF Nph cKO) **(C)** were injected or not with 400µl pristane; 6 mo later splenic B cell subsets **(A-C)** and BM B cell precursors **(D, E)** were analyzed by flow cytometry. For gating strategy of B cell populations see *Methods*. Graphs summarize data from two to three independent experiments, *Baff^fl/fl^* naive, N=8; *Baff^fl/fl^* pristane, N=18; *Baff^fl/fl^ zDC^Cre+^* naive, N=3; *Baff^fl/fl^ zDC^Cre+^* pristane, N=10; *Baff^fl/fl^ Cx3cr1^Cre+^* naive, N=4; *Baff^fl/fl^ Cx3cr1^Cre+^* pristane, N=12; *Baff^fl/fl^ Mrp8^Cre+^* naive, N=2; *Baff^fl/fl^ Mrp8^Cre+^* pristane, N=9. Statistics were performed by one-way ANOVA with Holm-Sidak method for multiple comparisons; *p<0.05, **p<0.01, ***p<0.001, ****p<0.0001.

We then investigated whether the absence of BAFF-producing MOs, DCs or Nphs affected splenic B cell levels in pristane-induced lupus-prone mice. Before pristane injection, B cell subsets in spleens of 2-3 mo old mice lacking BAFF from DCs, MOs and Nphs were not significantly different from control *Baff ^fl/fl^* mice, although a trend toward a decrease in FO and MZ B cells was observed in BAFF cDC cKO mice (**Supplementary Figure S3A** and data not shown). Thus, the lower BAFF serum levels observed in BAFF cDC cKO mice and BAFF MO cKO mice before pristane injection (**Figure 6**) did not affect mature splenic B cells homeostasis, confirming previous findings using BM chimeras (50). In the long-term (6 mo) pristane experiments we left some mice untreated (naïve) as controls. In contrast to the younger mice, these 8-9 mo old naïve mice lacking BAFF in cDCs had lower numbers of mature B cells and PCs than untreated *Baff ^fl/fl^* mice (**Figure 8A**). Specifically, when BAFF was missing in cDCs, spleens had lower numbers of FO, MZ and MZP B cells, while newly formed T2 and T1 B cells were unchanged (**Figure 8A**, *lower panels*). Like older naive mice, pristane-treated BAFF cDC cKO mice had lower mature B cell and PC numbers compared to *Baff ^fl/fl^* mice (**Figure 8A** and **Supplementary Figure S3B**). However, in addition to mature B cells, pristane-treated BAFF cDC cKO mice had fewer T2 B cells and CD11c^+^ B cells than *Baff ^fl/fl^* mice, indicating that in pristane-treated mice the lack of BAFF derived from cDCs more broadly affected B cell numbers.

In contrast to mice lacking BAFF in cDCs, naïve 8-9 mo old BAFF MO cKO mice had similar numbers of splenic B cells as naïve *Baff ^fl/fl^* mice (**Figure 8B**). However, BAFF from MOs was required to maintain normal mature B cells numbers after pristane injection (**Figure 8B** and **Supplementary Figure S3B**). Lack of BAFF from Nphs did not significantly affect FO or MZ B cells in either 8-9 mo naïve mice or pristane-treated mice (**Figure 8C** and **Supplementary Figure S3B**). Interestingly, pristane-treated BAFF Nph cKO mice had increased numbers of GC B cells compared to *Baff ^fl/fl^* mice, suggesting that Nph-derived BAFF may affect the generation of GC B cells, induced by pristane (**Figure 8C**).

Since cDC-derived BAFF contributed to systemic BAFF levels in naive mice independently of age, and it was required to maintain splenic mature B cells only in older mice, we examined whether BAFF produced by cDCs played a role in mature B cell homeostasis in the BM. BMs from either younger or older, as well as pristane-treated, BAFF cDC KO mice had lower numbers of mature B cells, but similar numbers of B cell precursors, compared to *Baff ^fl/fl^* mice (**Figure 8D** and **Supplementary Figure S4**). MO-derived BAFF also contributed to mature B cell homeostasis in the BM of 2-3 mo old mice (**Supplementary Figure S4**). In long-term experiments, the absence of BAFF-producing MOs affected BM mature B cells only after pristane treatment, similarly to what we observed in the spleen (**Figure 8E**). BMs from either younger naïve or pristane-treated BAFF MO cKO mice had lower numbers of Pro-B cells, suggesting a possible role for MO-BAFF, but not cDC-BAFF, in earlier stages of B cell development (**Figure 8E** and **Supplementary Figure S4**). In contrast, Nph-derived BAFF did not contribute to mature B cell homeostasis in the BM of pristane treated mice (data not shown).

Taken together our data from **Figures 6**, **8** highlight how different myeloid BAFF sources play overlapping, but also, specific roles in supporting systemic BAFF and B cell homeostasis in an inducible lupus-like setting. First, in younger mice cDC-derived and MO-derived BAFF are required to maintain systemic BAFF levels and mature B cell homeostasis in the BM. Second, BAFF produced by cDCs is also required to sustain systemic BAFF and mature B cell homeostasis in the spleen and BM of older mice. Third, after induction of autoimmunity in PIL, cDCs and MOs are major cellular BAFF sources driving the up-regulation of systemic BAFF and sustaining mature B cells in the spleen and BM. Finally, Nph-derived BAFF does not play a major role in splenic mature B cell homeostasis but contributes to the increase in pristane-induced systemic BAFF and regulates GC B cell numbers during PIL.

### Nphs, MOs and cDCs are all BAFF sources required for the induction of anti-SmRNP IgG autoantibodies in pristane–induced lupus mice

**Figure 9:**
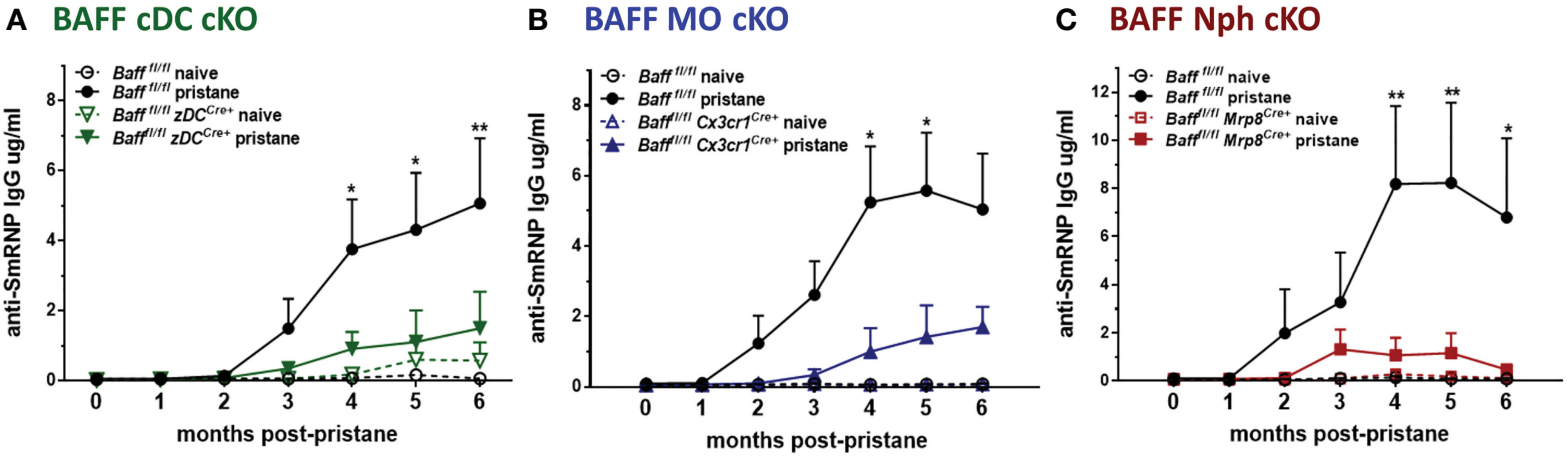
BAFF from Nphs, MOs and cDCs is required for the induction of pristane–induced anti-smRNP antibodies. *Baff^fl/fl^*, *Baff^fl/fl^ zDC^Cre+^* mice (BAFF cDC cKO) **(A)**, *Baff^fl/fl^ Cx3cr1^Cre+^* mice (BAFF MO cKO) **(B)** and *Baff^fl/fl^ Mrp8^Cre+^* mice (BAFF Nph cKO) **(C)** were treated or not for 6 mo with 400µl pristane. At the indicated time points sera were collected and anti-SmRNP Abs were measured by ELISA. Graphs summarize data from two to three independent experiments with three or more mice per group (*Baff^fl/fl^* naïve, N=4-6; *Baff^fl/fl^* pristane, N=10-19; *Baff^fl/fl^ zDC^Cre+^* naïve, N=3; *Baff^fl/fl^ zDC^Cre+^* pristane, N=9; *Baff^fl/fl^ Cx3cr1^Cre+^* naïve, N=4; *Baff^fl/fl^ Cx3cr1^Cre+^* pristane, N=10; *Baff^fl/fl^ Mrp8^Cre+^* naïve, N=2; *Baff^fl/fl^ Mrp8^Cre+^* pristane, N=9). Statistics were performed using 2-way ANOVA with Tukey’s multiple comparison test; *p<0.05, **p<0.01. Statistics shown are comparison between pristane treated *Baff^fl/fl^* mice and pristane treated BAFF cKO mice.

Finally, we tested whether the decrease in systemic BAFF levels in BAFF cDC cKO mice and BAFF MO cKO mice affected the auto-Ab responses induced by pristane. Anti-SmRNP IgG auto-Abs were induced in *Baff ^fl/fl^* mice and peaked 4-6 mo after pristane injection; however, when BAFF was absent from either cDCs or MOs, the auto-Ab responses were substantially reduced (**Figures 9A, B**). Surprisingly, even though systemic levels of BAFF, mature B cells and PCs were normal in mice lacking Nph-BAFF, pristane-induced anti-SmRNP IgG was significantly lower in these mice compared to *Baff ^fl/fl^* control mice (**Figure 9C**). Serum levels of auto-Abs were not reduced in all the corresponding control Cre mice (data not shown). These data suggest that although BAFF-producing Nphs may have a more localized effect on B cells, they still play a significant role in promoting auto-Ab production in this model. In conclusion, BAFF produced from three myeloid subsets- cDCs, MOs. and Nphs- is required for the induction of auto-Abs in a lupus-like autoimmune model.

## Discussion

BAFF plays an essential role in the pathogenesis of SLE and other autoimmune diseases. Since the anti-BAFF Ab, Belimumab, was approved for the treatment of SLE, several new drugs targeting different forms of BAFF or the BAFF/APRIL axis are being developed (26, 29, 30). However, still little is known about how, when, and where BAFF regulates the development of autoreactive B cells (15, 27–29, 51). In the current study, using novel BAFF-RFP reporter mice, BAFF cKO mice and both spontaneous as well an inducible mouse lupus-models, we defined cDC-, MO- and Nph-BAFF as major BAFF sources regulating specific B cell niches. Each of these cellular sources was required for the full development of SLE auto-Ab responses in an inducible lupus-model.

We and others have identified BAFF produced by cDCs and MO-derived DCs as essential for B cell and Ab responses in secondary lymphoid organs (10, 11, 42, 52, 53).

Previous studies described peripheral myeloid cells expansion in *Tlr7* Tg mice but did not address BAFF expression (36, 45). Utilizing heterozygous BAFF-RFP reporter mice, which when crossed with *Tlr7.1* Tg or *Sle1* mice spontaneously developed a lupus-like phenotype, we found that both mouse models had expanded splenic BAFF^+^-Nph, -MOs and -cDCs. Furthermore, BAFF expression was up-regulated on a per cells basis in inflammatory MOs in both *Tlr7* Tg and *Sle1* mice, while CD8^-^ DCs increased BAFF expression only in the *Tlr7* Tg model. In addition to myeloid cells, T cells upregulated BAFF expression and BAFF^+^ T cells were expanded in both models. Although BAFF expression in T cells has been suggested in CD4 T cells in human SLE (54–56), our results are the first clear demonstration that BAFF expression in T cells may be as high as in myeloid cells in mouse lupus. BAFF^+^ B cells were expanded in the *TLR7* Tg mice, and BAFF expression per cell increased in mature B cell subsets. BAFF^+^ PCs and other BAFF^+^ B cells in the peritoneal cavity were expanded in PIL These findings agree with other studies linking the elevated expression of BAFF in B cells in mouse lupus-models and SLE patients and the production of auto-Abs (57, 58). The ability of B cells to produce autocrine BAFF and sustain their own survival could play an important role in Type-I IFN and TLR7-driven autoimmunity. The exact contribution of BAFF^+^ T or B lymphocyte populations in the development or exacerbation of autoimmunity needs to be further explored.

Very few studies have investigated the production of tissue or serum BAFF in the PIL model (59–62). Here, we determined that serum BAFF is up-regulated and peaks at one month post-pristane injection. In PIL BAFF was elevated in multiple myeloid cell populations similar to the two spontaneous models. The reduced transcription of BAFF in splenic Nphs from pristane-treated BAFF-RFP mice, as well as BAFF-RFP *Sle1* mice may be due to Nph activation as in a *Salmonella* infection model (11). Nphs constitutively store high levels of BAFF that is released upon activation and drives PC responses (63, 64). Pristane-induced expansion of BAFF-expressing Nphs, MOs and DCs, similar to the spontaneous lupus models, was associated with increased BAFF serum levels, PC’s expansion, and induction of auto-Abs. MO recruitment, IFN-I induction and auto-Ab production in PIL all require TLR7/Myd88 signaling (40, 65). The inhibition of TLR7-mediated activation and BAFF production by DCs protects from PIL (60). Thus, in a lupus model where the TLR7/IFN-I axis is implicated in the development of autoimmunity, BAFF-producing DCs, MOs and Nphs each contribute to the induction of auto-Abs.

All pristane-treated mice in this study developed lipogranulomas (not shown), indicating disease development (65). However, we could not conclusively determine quantitative differences between the groups on lipogranuloma formation. The PIL model on the B6 background has some limitations (39); pristane-treated B6 mice do not develop robust anti-chromatin auto-Ab responses and develop very mild glomerulonephritis (60, 62). Even though renal function was not examined in this study, previous reports have suggested that all three myeloid BAFF sources studied here may be involved in the regulation of IgG deposits in the kidney (60, 62, 66). While BAFF overexpression can efficiently amplify underlying predisposition to clinical disease, it may not be sufficient to drive the development of severe glomerular pathology in mice (1, 67, 68). Therefore, it will be of future interest to test whether the deletion of any of these specific myeloid BAFF sources could improve renal disease in other more robust lupus-prone mice models.

While all three myeloid BAFF sources were required for the induction of auto-Abs, each subset affected systemic BAFF levels and mature B cells pools differently. For example, while either MO-BAFF or cDC-BAFF was required to maintain systemic BAFF levels and mature B cell homeostasis in the BM of younger mice, only BAFF produced by cDCs was required for mature B cell homeostasis in older mice. DC-BAFF was required to sustain mature splenic B cells only in older untreated or pristane treated mice, while MO-BAFF affected the B cell pool only after the induction of autoimmunity. Further studies are needed to assess the involvement of myeloid-derived BAFF in regulating B cell homeostasis during aging, which could also play a role in the development of autoimmunity (69).

Nph-BAFF, in contrast to cDC-BAFF and MO-BAFF, did not play a major role in determining systemic BAFF levels or FO and MZ B cell homeostasis. Using long-term neutrophil-depletion, Coquery et al. reported that Nphs contribute to excess serum BAFF levels and B cell responses in a congenic lupus-prone mouse model (70). In contrast, in PIL mice we found that Nph-BAFF was required for auto-Ab production, but only partially supported normal BAFF serum levels. This difference could be due to differences in the two models used or could reflect the possibility that Nph-depletion independently affects other BAFF-sources. Surprisingly, conditional BAFF depletion from Nphs increased GC B cell numbers in PIL, implying that Nph-BAFF may play a role in regulating GC formation. Consistent with our finding, Nph depletion early in lupus resulted in a striking acceleration in the onset of lupus renal disease and GC formation in secondary lymphoid organs (71). Given the complex role of Nphs in lupus (66, 72), the effect of Nph-BAFF on GC, serum BAFF and auto-Abs in PIL mice can be interpreted in the context of different disease kinetics and the localized effects of Nph-BAFF in specific B cell niches. For example, although Nphs play a major role in extrafollicular (EF) humoral responses, they can also localize in T-cell zones and interact with GC B cells in lupus-prone mice (70, 73). Both EF and GC-dependent B cell activation pathways contribute to pathogenic auto-Ab production in SLE, and clinical data suggest that these two pathways maybe responsible for some heterogeneity in human SLE patients (28). Thus, a possible explanation for our findings is that Nph-derived BAFF may skew toward EF B cell responses, away from GC formation. Our findings are consistent with a growing number of studies suggesting that a BAFF-producing Ly6G^lo^ Nph subset supports Ag-specific humoral responses in lupus as well as after microbial infections (74–76). Thus, our data suggest that Nphs and Nph-BAFF may play a more pathogenic role in the development of autoreactive B cells in lupus and highlight the importance of defining the specific context where Nphs interact with B cells.

Reeves et al. found that in PIL mice, Ly6C^hi^ inflammatory MOs are the primary source of IFN-I (41, 77). IFN-I sustains the chronic inflammatory response that leads to autoimmunity, by promoting Ly6C^hi^ inflammatory MOs continuous recruitment to the peritoneum and lymphoid tissues, and blocking their ability to differentiate into Ly6C^lo/int^ MO/Mphs (41, 77). Ly6C^lo/int^ MO-derived Mphs are mature stages of MO differentiation, more phagocytic and less inflammatory than Ly6C^hi^ MO, they play a major role in the resolution of inflammation, tissue repair and homeostasis (47–49). Here we found that DC-BAFF and MO-BAFF, but not Nph-BAFF, were required to maintain high expression levels of the inflammatory marker Ly6C on MOs in PIL mice. Consistent with our results, BAFF receptors on human MOs are associated with the release of inflammatory mediators, while suppressing phagocytosis and migration *in vitro* (78–80). Furthermore, lupus and Sjogren’s syndrome patients have abnormally elevated expression of BAFF receptors on MOs (54, 81). Thus, an interesting possibility is that DC-BAFF and MO-BAFF contribute to the amplification of the inflammatory cascade by promoting inflammatory Ly6C^hi^ MOs and restraining their differentiation into more mature MOs (47–49). Future research could test whether BAFF directly induces production of IFN-I or other cytokines in MOs creating a positive feedback circuit, and whether BAFF regulation of MOs may play a role in the exacerbation of lupus autoimmunity induced by chronic inflammatory responses (77, 82).

As in the PIL model where IFN-I drives the autoimmune response, the high serum BAFF levels we observed in *Tlr7.1* Tg mice was likely due to the substantial increase in IFN-I induced by TLR7 overexpression (31, 32, 36). A similar correlation between up-regulation of TLR7 and high BAFF serum levels occurs in immune thrombocytopenia, another autoimmune disorder (83). Both TLR7 and IFN-I signaling, particularly in myeloid populations, are strong drivers of lupus-like pathology and auto-Ab production in *Sle* mice (84, 85). However, previous studies only found higher BAFF levels in the serum when crossing *Sle1* mice with an autoimmune accelerator that overexpresses TLR7, resulting in severe systemic autoimmunity and kidney disease (38, 86). Thus, the increase in systemic BAFF in *Sle1* mice, which has been previously overlooked, further supports a role for BAFF in the induction of autoreactive B cells and auto-Abs in spontaneous lupus-like disease models (14, 15). Increases in systemic BAFF levels correlate with disease severity and auto-Ab responses in other lupus-prone mice like MRL^lpr/lpr^ mice and NZBWF1 (20, 70, 87), as well as in SLE patients (22–24, 88–90).

Humoral autoimmune responses in our parental lines were consistent with those described by us and others in *Tlr7.1* Tg mice (31–33, 45), and *Sle1* mice (35, 38). Notably, the induction of auto-Abs and the increase in PC numbers in BAFF-RFP *Tlr7.1* Tg mice and BAFF-RFP *Sle1* mice, which had only one functional BAFF allele and reduced mature B cells, was comparable to that of their parental lines. Similarly, high PC numbers and some auto-Abs were induced in BAFF-RFP mice also after pristane-induced autoimmunity. In autoimmune settings transitional/newly formed (NF) B cells may become more responsive to BAFF and play a role in the development of autoimmunity including SLE (28, 33, 44, 91–94). Since, NF B cells can differentiate in PCs and produce IgG Abs (9, 28, 33, 91, 92), it is possible that in the BAFF-RFP mice NF B cells contributed to auto-Abs responses in the lupus-prone models.

Despite the redundancy in the BAFF system, depletion of BAFF in anyone of the three myeloid cell types, DCs, MOs or Nphs was sufficient to ameliorate humoral autoimmunity. However, each BAFF source has a different role in regulating BAFF serum levels and seems to sustain mature B cells acting through different mechanisms. Consistent with our results, a few studies have pointed out a possible involvement of MO- and DC-BAFF in human SLE (95, 96). IFNα and SLE-immune complexes (IC) induce BAFF production and mobilization by blood MOs and DCs in SLE-patients with active disease, but not in healthy individuals or SLE patients with non-active disease (95). Serum from SLE patients *via* IFN-I instructs MOs to differentiate into DCs, which in turn mediate IgG-plasmablast differentiation *via* BAFF and IL-10 (96). Also, recent studies in mice and humans have highlighted how inflammatory MO-derived DCs and the plasticity of MOs play a critical role in mediating SLE autoimmunity and pathogenesis (77, 82, 97, 98). In addition, specific monocyte/DC signatures related to clinical disease, type I IFN signatures and responsiveness to treatments have been identified in Lupus patients (99, 100). Thus, it would be interesting to see whether BAFF expression correlates with different myeloid cell-signatures in pathological conditions and the ability to respond to BAFF-neutralizing agents. It is possible that some of the BAFF heterogeneity in SLE patients may reflect variable responses to inflammatory signals that induce BAFF in different subsets/locations (27, 28, 101). Our data, suggest that specific BAFF sources and B cell niches, implicated in the development of autoreactive B cells, may be important to consider when designing new lupus treatments (3, 19, 28, 51). In a recent study, BAFF gene silencing has effectively improved disease outcome in experimental SLE (102). DC- and MO-targeting and new technologies are starting to show promising results in autoimmune diseases (103, 104). Thus, targeting upstream signals of BAFF production by myeloid cell populations, or targeting BAFF in specific myeloid cells or lymphoid compartments, could be helpful in future lupus therapies (105).

## Data availability statement

The original contributions presented in the study are included in the article/**Supplementary Material**. Further inquiries can be directed to the corresponding author.

## Ethics statement

The animal study was reviewed and approved by University of Washington Institutional Animal Care and Use Committee.

## Author contributions

Conceptualization, DG, EC, NG. Data curation, DG, RK, KD. Formal analysis, DG, RK. Funding acquisition, EC, NG, DG, KE. Investigation, DG, RK, KD. Methodology, DG, RK. Project Administration, DG, NG, EC. Resources, EC, NG. Supervision, DG, NG, EC. Visualization, DG. Writing – original draft, DG. Writing – review and editing, DG, EC, KE, RK. All authors contributed to the article and approved the submitted version.
