# Supplementary material for: B cell-activating factor (BAFF) from dendritic cells, monocytes and neutrophils is required for B cell maturation and autoantibody production in SLE-like autoimmune disease
Source: Front Immunol. 2023 Feb 27;14:1050528. doi: 10.3389/fimmu.2023.1050528 (PMC10009188; doi:10.3389/fimmu.2023.1050528)
Supplement: Supplementary file 1 [file DataSheet_1.pdf]

Supplementary Fig. S1

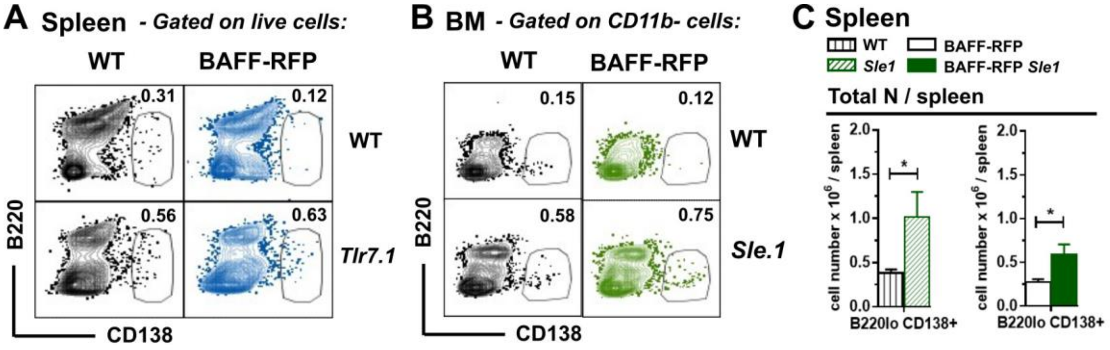

Supplementary Figure S1. Induction of PCs in 6-10 mo old BAFF-RFP *Tlr7.1* mice and BAFF-RFP *Sle1* mice. Spleens (**A** and **C**) and BM (**B**) from WT and BAFF-RFP (BAFF-RFP$^{+/-}$) mice (**A-C**), *Tlr7.1* and BAFF-RFP *Tlr7.1* mice (**A**), *Sle1* and BAFF-RFP *Sle1* mice (**B** and **C**) were harvested and analyzed by flow cytometry for B220^{lo} CD138^{+} plasma cells (PCs) numbers. **A** and **B**, show representative dot plots of PCs in spleens of *Tlr7.1* genotypes and BM of *Sle1* genotypes, respectively. In **A** and **B** numbers indicate % of BAFF-RFP^{+} cells. **C**, shows increased PCs numbers in spleens from *Sle1* and BAFF-RFP *Sle1* mice compared to WT and BAFF-RFP respectively. In **C**, results are shown as bar graphs (mean ± SEM) summarizing data from three independent experiments (WT and *Sle1* mice: N=7-9; BAFF-RFP, BAFF-RFP *Tlr7.1* and BAFF-RFP *Sle1*: N=9-13). Statistics were performed using two-tailed unpaired Student t test * $p<0.05$.

Supplementary Fig. S2

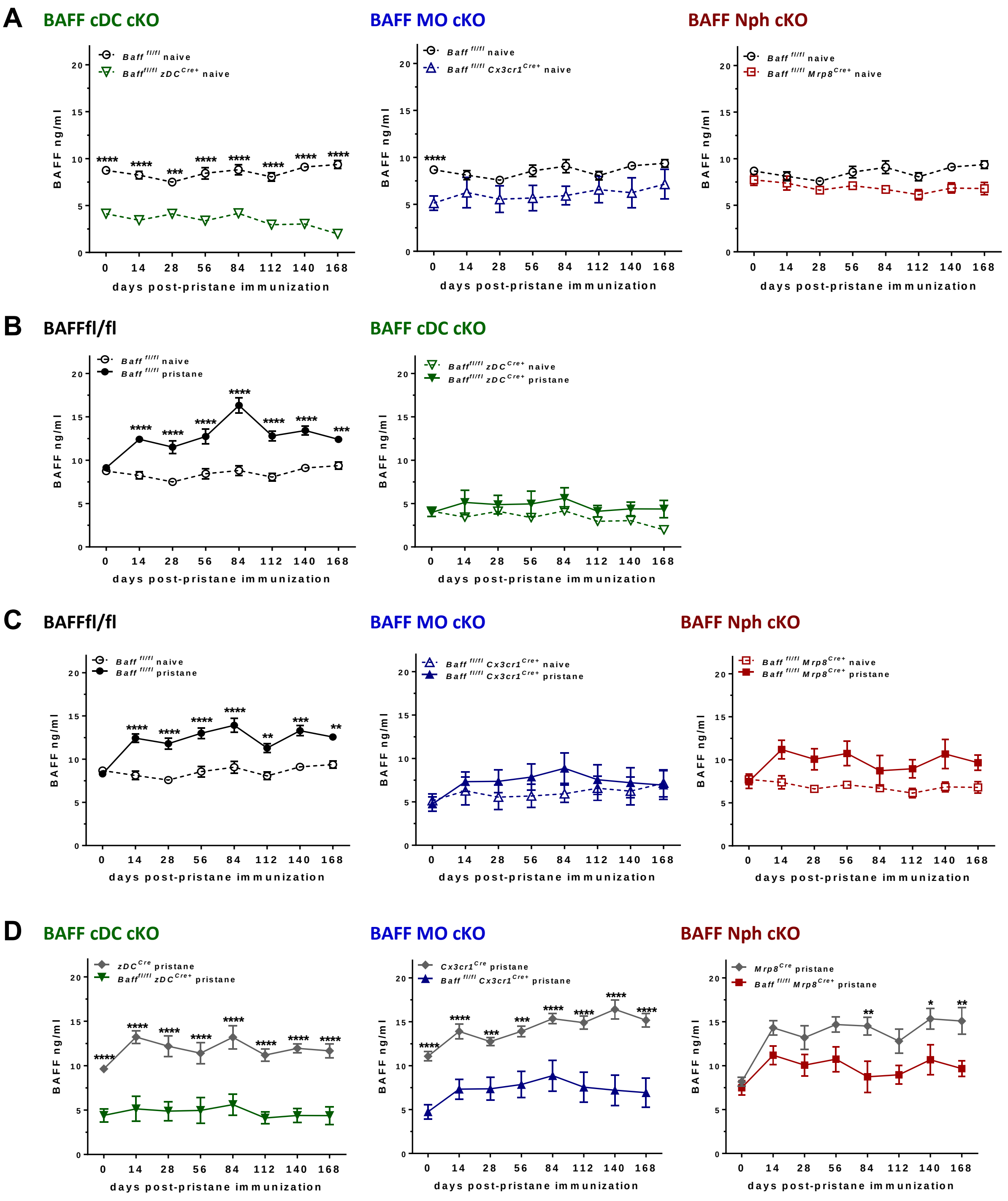

Supplementary Figure S2. BAFF serum in naïve and pristane-treated BAFF cDC cKO, BAFF MO cKO and BAFF Nph cKO mice. *Baff$^{fl/fl}$* mice (**A-C**), *zDC^{Cre+}* mice (**D**), BAFF cDC

cKO mice (*Baff*$^{fl/fl}$ *zDC*$^{Cre+}$) (**A, B, D**), *Cx3cr1*$^{Cre+}$ mice (**D**), BAFF MO cKO mice (*Baff*$^{fl/fl}$ *Cx3cr1*$^{Cre+}$) (**A, C, D**), *Mrp8*$^{Cre+}$mice (**D**) and BAFF Nph cKO mice (*Baff*$^{fl/fl}$ *Mrp8*$^{Cre+}$) (**A, C, D**) were treated (**B-D**) or not (**A**) for 6 mo with 400µl pristane. **A-D**, at the indicated time points sera were collected and BAFF levels were measured by ELISA. **A**, serum BAFF levels in naïve *Baff*$^{fl/fl}$ and BAFF cKO mice over a 6 mo time period. **B** and **C**, serum BAFF levels comparing naive vs. pristane-treated *Baff*$^{fl/fl}$ and BAFF cKO mice. **D**, serum BAFF levels in treated BAFF cDC cKO, BAFF MO cKO and BAFF Nph cKO compared to their relative Cre controls, e.g. *zDC*$^{Cre+}$, *Cx3cr1*$^{Cre+}$and *Mrp8*$^{Cre+}$mice, respectively. Graphs summarize data from two to three independent experiments (*Baff*$^{fl/fl}$ naïve, N=6-18; *Baff*$^{fl/fl}$ pristane N=6-21; *zDC*$^{Cre+}$pristane N=5-12; *Baff*$^{fl/fl}$ *zDC*$^{Cre+}$naïve, N=4-18; *Baff*$^{fl/fl}$ *zDC*$^{Cre+}$pristane, N=6-14; *Cx3cr1*$^{Cre+}$pristane, N=10-13; *Baff*$^{fl/fl}$ *Cx3cr1*$^{Cre+}$naïve, N=4-22; *Baff*$^{fl/fl}$ *Cx3cr1*$^{Cre+}$pristane, N=13-18; *Mrp8*$^{Cre+}$, N=5; *Baff*$^{fl/fl}$ *Mrp8*$^{Cre+}$naive N=2-12; *Baff*$^{fl/fl}$ *Mrp8*$^{Cre+}$pristane N=9-10). Statistics were performed using 2-way ANOVA with Tukey's multiple comparison test. * $p<0.05$, ** $p<0.01$, *** $p<0.001$, **** $p<0.0001$.

Supplementary Fig. S3

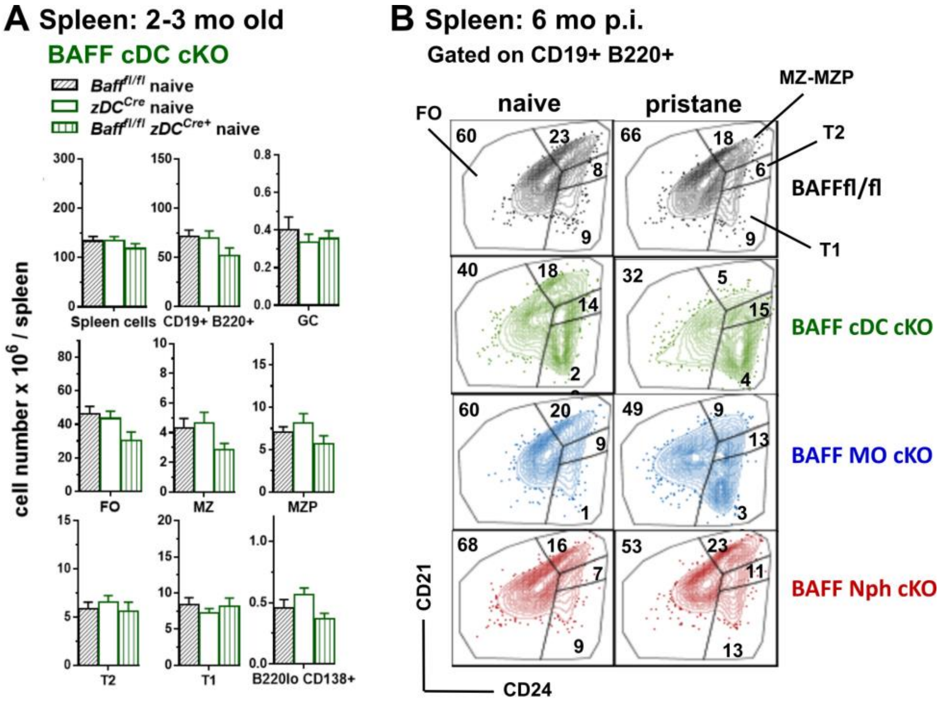

Supplementary Figure S3. Splenic B cell subsets in 2-3 mo old BAFF cDC cKO mice and 8-9 mo old BAFF cDC cKO, BAFF MO cKO and BAFF Nph cKO naïve and pristane-treated mice. A, Spleens from naïve 2-3 mo old *Baff*$^{fl/fl}$, *zDC*^{Cre}, *Baff*$^{fl/fl}$ *zDC*$^{Cre+}$ mice (BAFF cDC cKO) were harvested, and splenic B cell subsets were analyzed by flow cytometry. Bar graphs summarizes data from three independent experiments, *Baff*$^{fl/fl}$, N=7; *zDC*^{Cre}, N=6; *Baff*$^{fl/fl}$ *zDC*$^{Cre+}$ naive, N=9. **B**, *Baff*$^{fl/fl}$, *Baff*$^{fl/fl}$ *zDC*$^{Cre+}$ mice (BAFF cDC cKO), *Baff*$^{fl/fl}$ *Cx3cr1*$^{Cre+}$ mice (BAFF MO cKO) and *Baff*$^{fl/fl}$ *Mrp8*$^{Cre+}$ (BAFF Nph cKO) were injected or not with 400µl pristane and 6 mo later splenic B cell subsets were analyzed by flow cytometry. **B**, Representative dot plots of B cell subsets (for more on gating strategy see *Methods*) from two to three independent experiments of data summarized in Figure 8.

Supplementary Fig. S4

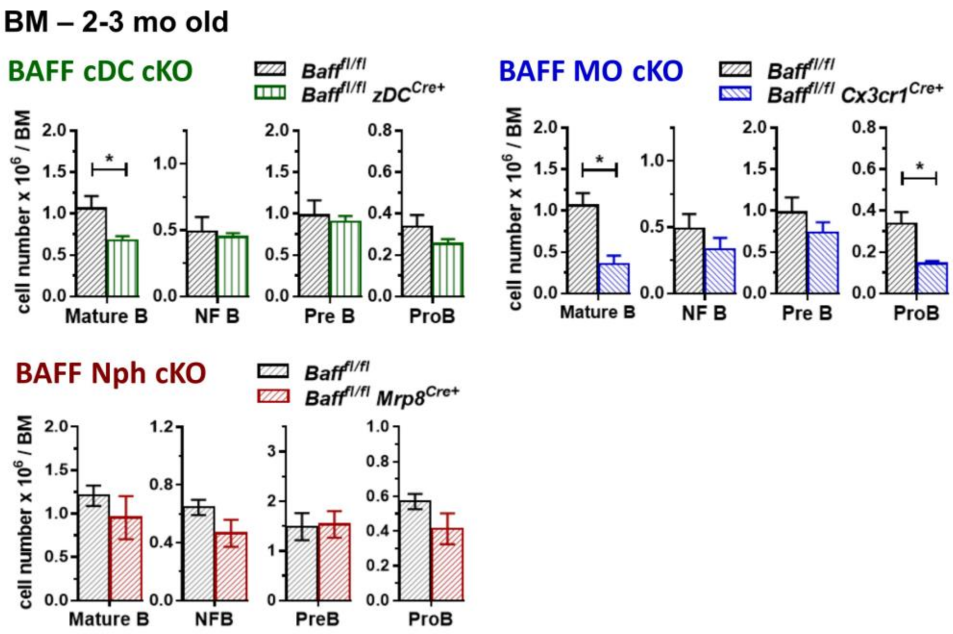

Supplementary Figure S4. BM B cells in naïve 2-3 mo old BAFF cDC cKO mice, BAFF MO cKO and BAFF Nph cKO naïve. BMs from 2-3mo old *Baff*$^{fl/fl}$ mice, *Baff*$^{fl/fl}$ *zDC*$^{Cre+}$ mice (BAFF cDC cKO: *upper left panel*), *Baff*$^{fl/fl}$ *Cx3cr1*$^{Cre+}$ mice (BAFF MO cKO: *upper right panel*) and *Baff*$^{fl/fl}$ *Mrp8*$^{Cre+}$ mice (BAFF Nph cKO: *lower left panel*) were harvested and B cell precursors were analyzed by flow cytometry. For gating strategy of BM B cell populations see *Methods*. Graphs show data from one experiment each, *Baff*$^{fl/fl}$ naive, N=3; *Baff*$^{fl/fl}$ *zDC*$^{Cre+}$ naive, N=5; *Baff*$^{fl/fl}$ *Cx3cr1*$^{Cre+}$ naive, N=3; *Baff*$^{fl/fl}$ *Mrp8*$^{Cre+}$naive, N=3. Statistics were performed by one-way ANOVA with Holm-Sidak method for multiple comparisons; * $p<0.05$.
